# Quantifying the seasonal drivers of transmission for Lassa fever in Nigeria

**DOI:** 10.1098/rstb.2018.0268

**Published:** 2019-05-06

**Authors:** Andrei R. Akhmetzhanov, Yusuke Asai, Hiroshi Nishiura

**Affiliations:** Graduate School of Medicine, Hokkaido University, Sapporo, Hokkaido, Japan

**Keywords:** Lassa haemorrhagic fever, Arenaviridae, multimammate rat, reservoir host, seasonality

## Abstract

Lassa fever (LF) is a zoonotic disease that is widespread in West Africa and involves animal-to-human and human-to-human transmission. Animal-to-human transmission occurs upon exposure to rodent excreta and secretions, i.e. urine and saliva, and human-to-human transmission occurs via the bodily fluids of an infected person. To elucidate the seasonal drivers of LF epidemics, we employed a mathematical model to analyse the datasets of human infection, rodent population dynamics and climatological variations and capture the underlying transmission dynamics. The surveillance-based incidence data of human cases in Nigeria were explored, and moreover, a mathematical model was used for describing the transmission dynamics of LF in rodent populations. While quantifying the case fatality risk and the rate of exposure of humans to animals, we explicitly estimated the corresponding contact rate of humans with infected rodents, accounting for the seasonal population dynamics of rodents. Our findings reveal that seasonal migratory dynamics of rodents play a key role in regulating the cyclical pattern of LF epidemics. The estimated timing of high exposure of humans to animals coincides with the time shortly after the start of the dry season and can be associated with the breeding season of rodents in Nigeria.

This article is part of the theme issue ‘Modelling infectious disease outbreaks in humans, animals and plants: approaches and important themes’. This issue is linked with the subsequent theme issue ‘Modelling infectious disease outbreaks in humans, animals and plants: epidemic forecasting and control’.

## 1. Introduction

Growing human population, urbanization and global warming increase the chance of human interaction with wildlife, resulting in elevated risk of zoonotic diseases. Despite numerous publications assessing the risk factors for viral spillovers, published studies have tended to miss out quantitative estimation of their frequency and characteristics and causal links among different host species. In this study, we provide quantitative analysis of external forcing of infection, as applied to Lassa haemorrhagic fever (LF), a widespread disease in West Africa. The proposed modelling framework can be further extended to other diseases with common characteristics and viewed as key in light of anticipated emergence of a novel infectious disease in the future—the ‘Disease X’ as it was recently coined by the World Health Organization.

LF is an acute haemorrhagic viral infection, caused by an enveloped, single-stranded RNA virus of the Arenaviridae family with a clear zoonotic origin [1–3]. The number of reported cases reaches about a thousand per year including a few hundreds of deaths, and one-third of cases experience severe post-infection complications (e.g. lifetime deafness). For these reasons, LF infection is recognized as a serious public health concern that necessitates future research on its pathogenicity and possible prevention.

LF virus was first discovered in 1969, but its presence can be traced back for centuries [4]. Current viral hotspots are focused on West African countries, namely, Nigeria, Guinea, Benin, Sierra Leone and Liberia. The animal reservoir of the virus is *Mastomys natalensis*, also known as multimammate mouse, which is a rodent species that is widespread in the region. Although the main transmission route remains zoonotic, i.e. animal-to-human, contracting the virus through exposure to contaminated excreta or secretions from rodents [5], another frequently reported route is human-to-human transmission, via contact exposure to the virus from the blood or bodily fluids of an infected person. The latter contributes to approximately 19% of all reported cases and is usually observed during nosocomial outbreaks [6]. Hence, the overall transmission pattern is primarily driven by environmental exposure to LF [3] rather than sustained human-to-human transmission chains, being different from Ebola virus disease [7]. Given the importance of the human–animal interface, we anticipate that environmental factors may be crucial driving forces contributing to the frequent recurrence of LF outbreaks.

Observed LF incidence exhibits a strong seasonal component: the number of reported cases is likely to rise in the first two to three months of the year, exceeding the baseline over the rest of the year several-fold. This phenomenon can be captured by implementing a time-varied probability of exposure in our model. Two distinct time periods characterize a low and high risk of exposure to the virus. Importantly, the model fit allows the probable time boundaries of each period to be determined.

Published estimates of the case fatality risk (CFR) from LF of 1–2% have been proposed for previously healthy populations without underlying comorbidities [8]. However, this rate varies considerably depending on the context, being for example 2–5% for hospital-treated cases and increased to 20–60% for laboratory-confirmed cases or during nosocomial outbreaks [2,9,10]. Because higher estimates of the CFR are likely due to underreporting and ascertainment bias, we are able to account for this observational matter and estimate an underreporting factor. Similarly to our recent work [11], we assume that fatal cases are certainly reported in the surveillance system throughout the whole year, while less severe, non-lethal cases are likely to be missed. Such variation may especially be the case during low-risk periods, when LF infections are not frequently reported.

The establishment and maintenance of a surveillance system inevitably face many difficulties in the case of LF reporting. A clinical diagnosis of LF infection is often challenging owing to the similarities with other common diseases seen in the region, i.e. malaria or typhoid fever [12,13]. On average, four out of five cases are asymptomatic [8]. Furthermore, post-mortem examinations to confirm LF infection as the cause of death are recognized as taboos in some areas of Nigeria [1]. All of these aforementioned factors contribute to the limited accuracy and completeness of the passively reported surveillance data.

Here, we offer a unified epidemiological model that consists of two parts [14]. The first part describes the process of generating the incidence data in humans. We derived the risk of exposure to the virus throughout the year. The second part employs the so-called susceptible–infected–recovered (SIR) model, which captures the transmission dynamics of the virus in rodents. Based on existing observations of rodent populations both in the field and in laboratory settings [15–17] (see the discussion on the difficulties of collecting data in Nigeria in [18]), we have captured the seasonality in the numbers of infected rodents. Using the first and second parts of the model, we also estimated the relative contact frequency of humans with infected animals throughout the year.

Our study encompasses the analysis of surveillance data on LF incidence in humans along with the transmission dynamics in the rodent reservoir. Using this modelling framework, we aimed to estimate the impact of environmental factors on the annual fluctuations in the risk of LF infection in humans. Similar methodological applications can be performed for other zoonotic diseases that involve well-identified wildlife animal hosts.

## 2. Results

First, we estimated two distributions from existing datasets in humans, i.e. the incubation period and the time from illness onset to death. We used a published dataset from a nosocomial outbreak in Jos, Nigeria, in 1970 [19], to determine the best-fit gamma distributions to be a mean of 12.8 days (95% credible interval (CrI): 10.7–15.0) and a standard deviation of 4.6 days (CrI: 2.8–6.6) for the incubation period, and a mean of 13.8 days (CrI: 10.8–17.0) and a standard deviation of 7.6 days (CrI: 5.0–10.6) for the time from illness onset to death (electronic supplementary material, appendix A and figure S1).

**Figure 1.**
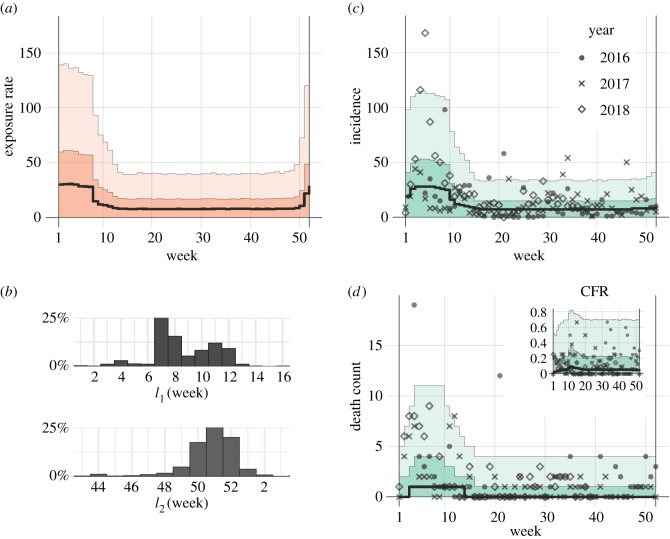
Temporal distribution of Lassa fever incidence in Nigeria, 2016–2018. (*a*) Fitted exposure rate as a function of the calendar week in a given year. (*b*) Posterior distribution for the time boundaries of high-/low-risk exposure periods. (*c*,*d*) Model fit to the observed data of new cases and fatal cases. Inset in (*d*) shows the expected case fatality risk (CFR). Solid black line indicates the median estimate, whereas light and dark shaded areas in (*a*,*c*,*d)* indicate 95 and 50% credible intervals for posterior estimates, respectively. (Online version in colour.)

By incorporating these estimates into our analysis of the incidence data (Material and methods section), weekly exposure rates of humans to LF were estimated. We used a model that rests on the renewal process of the viral exposure in humans, accounting for the time delay due to the incubation period and the time from illness onset to death. The formulation resulted in two Poisson- and binomially distributed likelihood functions for describing the LF incidence and mortality, respectively (electronic supplementary material, appendix B). We identified two distinct periods of a year which were separately analysed owing to considerably different levels of observed transmission activity: (i) a low-risk period with a weekly exposure rate of 6.4 (CrI: 0–32.5; maximum-likelihood estimate (MLE): 9.6) lasting from week 9 (26 February to 4 March in 2018, CrI: 5–13) to week 50 (10 December to 16 December in 2018; CrI: 47–52), and (ii) the rest of the year, characterized by a higher risk of contracting the virus, with the average weekly exposure rate reaching 24.7 (CrI: 0–111.7; MLE: 38.1), which exceeded the low-risk period by approximately four times. The CFR was estimated at 4.9% (CrI: 0–54.4; MLE: 8.9%). If we assume the unbiased value of the CFR at 2%, the reporting coverage can be estimated to be as low as 40%. The overlaid incidence data and variations in the parameter estimates are shown in figure 1. For comparison, an analogous model with a single (constant) exposure rate yielded a less plausible fit to the data (Akaike information criterion (AIC) values: 3679.2 for a single- and 2877.2 for a two-period model).

Subsequently, we assessed whether a seasonal increase in the number of infected rodents can solely explain the increase in the incidence of LF infections in humans in the high-risk period. Using the dynamic transmission model for LF infection in rodents (Material and methods section; electronic supplementary material, figure S2), the number of infected animals was predicted to reach its maximal level in the middle of May, while the minimal level was observed in December (electronic supplementary material, figure S3). The LF incidence in humans which is thought to correlate with the LF level of infection in rodents controversially peaks in the first two to three months of the year. During that period, the number of infected rodents was smaller than for the rest of the year. We therefore propose that a factor other than seasonal fluctuations in the number of infected rodents may play a role in driving LF seasonal epidemics.

**Figure 2.**
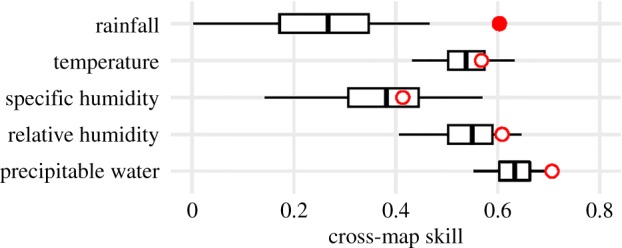
Cross-map causality for shared seasonality of environmental variables with the Lassa fever incidence. Red circles show the unlagged cross-map skill. Box-plots show null distributions for cross-map skill expected from random surrogate time series that share the same seasonality as the true environmental variable. The single filled circle indicates that the measured causality is significantly better than the null expectation (*p* < 0.01). As for the use of convergent cross-mapping, please see literature [20,21]. (Online version in colour.)

To search for such a factor, we analysed the correlative power of the LF incidence rates and climatological variables, such as rainfall, temperature, relative humidity, specific humidity and precipitable water. We found that only the rainfall seasonal pattern was significantly correlated with the seasonal incidence of LF in humans (*p* < 0.01, figure 2). This was also confirmed by the start and end times of the high/low exposure periods, which approximately coincide with the start and the end of the rainy season (cf. figure 1*b*; electronic supplementary material, figure S4). Therefore, an event associated with rainfall patterns contributes to the incidence of LF in humans, e.g. this could be the change in seasons and seasonal migration or a change in the behaviour of the rodents.

**Figure 3.**
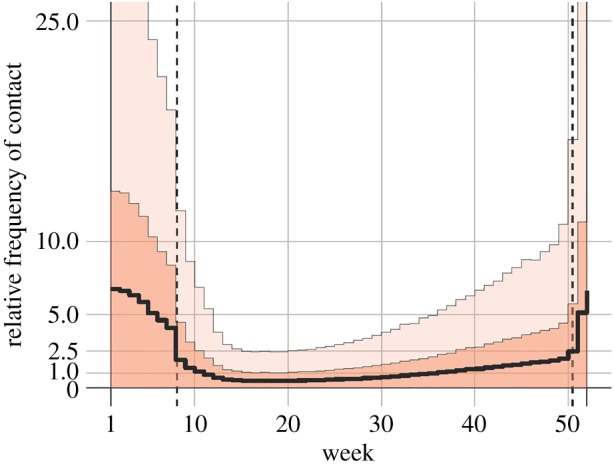
Contact frequency of humans with infected rodents. Dashed vertical lines show the time boundaries separating the high-risk period from the low-risk period. The average contact frequency in the low-risk exposure period was set to 1. Solid black line indicates the median estimate, whereas light and dark shaded areas indicate 95 and 50% credible intervals for posterior estimates, respectively. (Online version in colour.)

Last, we calculated the relative frequency of contact of humans with infected rodents using the ratio of the estimated weekly exposure rate to the density of infected rodents predicted by our transmission model (figure 3). After assigning the average frequency of contact in the low-risk exposure period as 1, we determined that the relative risk of human exposure was 5.3 times higher on average during the high-risk exposure period. The maximal value of 6.7 was identified in the first week of the year.

## 3. Discussion

This study analysed the surveillance-based incidence data of LF in Nigeria from 2016 to 2018 and identified two different (high- and low-)risk periods. The high-risk period spanned the last month of the year to the second to third month of the year. The relative risk of acquiring LF infection during the high-risk period was five times greater than during the rest of the year. In our search for a possible explanation, we identified that the rainfall pattern was negatively and highly correlated with LF incidence. The rainfall does not affect the transmissibility of the virus directly, but it is noteworthy that rodents migrate to within close proximity to human settlements to breed and hibernate during the dry season. This in turn leads to an increase in the contact rate of humans with rodents, and as a consequence, a higher probability of acquiring LF infection.

Fichet-Calvet & Rogers [22] previously showed that the rainfall pattern was the main single abiotic factor contributing to LF. The migration of rodents was notified as a main factor in another report [23]. Our findings confirm the importance of the combined effect of these two factors on the seasonality of LF epidemics and offer a quantitative estimation of their strength, which has never to our knowledge been done in the past. When the rainy season ends in November, the breeding season for rodents starts about two months later. At that time, newly born offspring and scarcity of food on the ground force mature rodents to approach human-occupied areas. Consequently, this may lead to a rise in the contact frequency between humans and infected rodents. This high-exposure frequency persists until the rainy season starts again the following year, at which point the rodents migrate back to the ground and we observe the subsequent decline in human cases of LF. These findings highlight the importance of seasonal ecology of animal hosts in explaining the seasonality of LF epidemics. Similar findings have been reported for many other diseases, not only for those of an epizootic nature, e.g. [24,25], but also for insect-vectored plant diseases [26]. This points to rather general applicability of our approach that may frame the spillovers of other pathogens with two epizootic transmission routes. An important achievement of the present study was that we were able to offer quantitative estimates of the impact of the two risk factors, rainfall pattern and migration of rodents, on the exposure probability of humans to LF infection.

This highlights the importance for preventive measures that aim to contain seasonal epidemics of LF to be specifically designed: (i) to control rodent populations and reduce the encounter frequency of rodents and humans, and (ii) to raise awareness among local residents. This can be envisioned as an eradication campaign, especially in rural areas with agronomic activities, and in public markets in urban areas, where rodents are frequently seen. Preventive measures may also include improved hygiene practices, hiding food from rodents during the night time, or designing educational campaigns that raise awareness of LF pathogenicity. The implementation of such programmes would be expected to lead to a decline in LF cases in the near future.

However, we were unable to measure the impact of awareness of the scale of epidemics using the available data. Even though occasional peaks in the number of recorded deaths may be indicative of periods of low awareness, our analysis of seasonal trends only showed that awareness probably does not involve a strict seasonal component. Nevertheless, the effect of awareness on the reporting of LF incidence may be indirectly evident from the geographical distribution of LF cases over the last 6 years (electronic supplementary material, figure S5). The geographical area with newly reported LF cases has expanded in the last few years, presumably because of better recognition of the epidemics and improved capability of the surveillance system.

Several technical limitations of our study must be noted. First, we did not consider a spatial component in our analysis which may involve geographical heterogeneity in reporting rates. Second, we used only the counts of suspected cases that included both laboratory-confirmed cases and cases that tested negative. However, the available dataset indicates that the majority of suspected cases involved specimens that tested negative. This becomes especially evident in low-risk exposure periods, and taking this into consideration in our analysis would further amplify the difference between high-risk and low-risk periods. Third, we did not account for temporal changes in the surveillance system and improvements in laboratory facilities for the detection of LF, both of which have been greatly improved in recent years. Fourth, we did not distinguish possible variations in population densities of rodents in habiting human houses and farm fields or, similarly, urban and rural areas.

In summary, we identified and quantified the effect of two factors that drive the seasonality of LF epidemics in Nigeria. Combining the fit of the mechanistic model to the observed counts of LF disease in humans with the predictive model of the transmission dynamics of LF in rodents, we quantified the annual change in relative contact frequency between humans and infected rodents. A high seasonal amplitude was identified, and our model indicated the first nine weeks of the season as the high-risk period for LF transmission.

## 4. Material and methods

### (a) Data collection

Data were routinely collected from the weekly epidemiological report of the Nigerian Centre for Disease Control (NCDC) for the period from 2016/week 4 to 2018/week 30. The extracted counts included newly suspected weekly cases for both positively and negatively testing specimens, as well as the number of fatal cases reported per week. For both cases and deaths, the corresponding week of the report represents the week in which the illness onset and death events occurred, respectively (i.e. fatal cases were not reported as a function of the week of illness onset). We did not use the available data for previous time periods (2012–2016) owing to irregular reporting and removal of reports from an official website of the NCDC. However, we provide all of the accumulated data as electronic supplementary material.

To access weekly data on climatological variables (i.e. precipitation, temperature, relative and specific humidity, precipitable water), we used publicly available gridded NCEP/NCAR Reanalysis data [27,28]. The chosen reference point was set to coordinates 6.75° N 6.25° E, located in Edo state, Nigeria. Historically, Edo state was characterized by a high prevalence of LF infection. It was also marked as highly hazardous for LF transmission owing to an average annual rainfall of 1786 mm [22]. Additionally, we used monthly data records over a longer time period (1901–2015) for the same location that were provided by another source: a publicly available dataset of the University of East Anglia Climatic Research Unit [29]. While the former was used for correlative analysis of rainfall patterns and available incidence data from the last 6 years, the latter was used only to generate the historical characteristics of rainfall, as shown in electronic supplementary material, figure S4.

### (b) Estimation of the incubation period and the time from illness onset to death

We fitted the distributions of the incubation period *f* and the time from illness onset to death *h*, which were essential for describing the epidemiological process, to gamma distributions. The analysed dataset included the cases and transmission events during a nosocomial outbreak in the Evangel Hospital in Jos, Nigeria, in 1970 (fig. 1 in [19]). In total, the number of described cases was 23 with probable time intervals of exposure, the date of illness onset and the date of death. To determine the best-fit parameters for both distributions, we applied a Markov chain Monte Carlo (MCMC) method in a Bayesian framework. The 95% credible interval (CrI) for each fitted parameter of the two distributions was determined as the 95% high-density interval [30].

### (c) Epidemiological modelling

First, we accounted for two transmission routes of LF infection, i.e. the animal-to-human route and the human-to-human transmission route. We specified a total exposure rate in weeks *w* by a variable *e_w_*. This could be written as the sum of the two rates: *e_w_* = *a_w_* + *h_w_*, where *a_w_* and *h_w_* are weekly exposure rates via animal-to-human and human-to-human transmission routes, respectively. An important estimate previously reported in the literature [6] indicated that *ca* 19% of all infections are attributed to the human-to-human transmission route, leading to the following requirement: *h_w_*/(*h_w_* + *a_w_*) = *r* = 0.19. We can then express the total exposure rate *e_w_* solely through the exposure rate *a_w_* such that: *e_w_* = *a_w_*/(1 − *r*). This expression was used in our framework to avoid a detailed modelling of the human-to-human transmission route. Owing to limited human-to-human transmission potential, we focused only on exposure to the virus through a contaminated environment.

Qualitatively, it has been noted that the incidence of infections is likely to rise in the first two to three months of each calendar year. We accounted for this in our model by incorporating seasonal variations in the weekly exposure rate $a_{w_{c}}$ as a function of the calendar week *w*_c_ (1 ≤ *w*_c_ ≤ 52). In its simplest form, the exposure rate $a_{w_{c}}$ was modelled by a two-level step function:

$$a_{w_{c}}(a_{\pm },\;l_{1,2})=\{\begin{array}{cc} a_{-}, & \mathrm{for}\;\;l_{1}\leq w_{c}\leq l_{2},\\ a_{+}, & \mathrm{otherwise}, \end{array}$$

where *l*_1,2_ represents two time boundaries separating a period of high exposure from a period of low exposure (1 ≤ *l*_1,2_ ≤ 52). We expected that *a*_+_ > *a*_−_.

Because all records in our dataset counted the weekly numbers *w* beginning from the first record, we assumed a functional dependence *w*_c_ = *w*_c_(*w*) in the following formulations.

These assumptions allowed us to define the epidemiological process in a concise form. Here, two Poisson processes are considered. First, each newly reported non-fatal case was dealt with as a result of previous exposure delayed by the length of the incubation period. This implied a Poisson process with the expected number of new cases *i_w_* at week *w* to be a result of the convolution of the exposure rate *a* and distribution of the incubation period *f*, multiplied by the chance of survival (1 − *q*) with *q* being the risk of death (or CFR):

$$E(i_{w}|\theta )=(1-q)\cdot \overset{w-1}{\underset{k=1}{\sum }}\frac{a_{w-k}(a_{\pm },\;l_{1,2})\times f_{k}}{1-r},$$

where ***θ*** = {*a*_±_, *q*, *l*_1,2_} is a set of model parameters.

In a similar manner, we introduced another Poisson process to describe the reporting of fatal cases. LF deaths result from exposure to the virus in previous weeks postponed by a probabilistically distributed time period between the exposure event and death. The latter was denoted by *g* and defined as a convolution of the incubation period *f* and the distribution of time from illness onset to death *h*. Then the expected number of fatal cases *d_w_* in week *w* was determined by a convolution of the exposure rate *a* and distribution *g*, multiplied by the risk of death *q*:

$$E(d_{w}\;|\;\theta )=q\cdot \overset{w-1}{\underset{k=1}{\sum }}\frac{a_{w-k}(a_{\pm },\;l_{1,2})\times g_{k}}{1-r}.$$

Then, the likelihood function for describing the total number of reports (i.e. cases plus deaths) was:

$$L_{i}(\theta \;|\{i_{w}+d_{w}\})=\underset{w}{\prod }\frac{{\left(E(i_{w}+d_{w}\;|\;\theta )\right)}^{i_{w}+d_{w}}\exp(-E(i_{w}+d_{w}\;|\;\theta ))}{(i_{w}+d_{w})!},$$

where {*i_w_* + *d_w_*} is a set of all available data records with a given independently reported incidence that includes both non-fatal and fatal cases, *E*(*i_w_* + *d_w_* | ***θ***) = *E*(*i_w_* | ***θ***) + *E*(*d_w_* | ***θ***).

The second likelihood function for describing the number of deaths was derived from the binomial sampling process:

$$\begin{array}{rl} L_{d}(\theta |\{i_{w}+d_{w},\;d_{w}\}) & =\underset{w}{\prod }\left(\begin{array}{c} i_{w}+d_{w}\\ d_{w} \end{array}\right)\\ & \;\times {\left(\frac{E(d_{w}\;|\;\theta )}{E(i_{w}+d_{w}\;|\;\theta )}\right)}^{d_{w}}{\left(1-\frac{E(d_{w}\;|\;\theta )}{E(i_{w}+d_{w}\;|\;\theta )}\right)}^{i_{w}}, \end{array}$$

where the terms in the first set of parentheses in the product denote the binomial coefficient, {*d_w_*}; on the left-hand side is a set of all available data records for weekly counts of deaths.

We first defined the model parameters ***θ*** by using a Bayesian approach and performing MCMC iterations. Second, we used the maximum-likelihood method to obtain point estimates. For the maximization procedure, we used the total (composite) likelihood of the form:

$$L_{\Sigma }(\theta \;|\;\{i_{w}+d_{w},\;d_{w}\})=L_{i}(\theta \;|\;\{i_{w}+d_{w}\})\;L_{d}(\theta \;|\;\{i_{w}+d_{w},\;d_{w}\}).$$

The likelihood *L*_Σ_ was maximized, respectively, to each parameter in ***θ*** by equating its first partial derivatives to zero.

### (d) Transmission dynamics of LF infection in rodents

To describe the transmission dynamics of LF infection in rodents, we used an SIR model (electronic supplementary material, figure S3 and more details in appendix C). The birth rate of rodents was defined by a periodic birth pulse function as suggested previously [31]. We used model parameter estimates previously derived in another field study on the same rodent species in Tanzania [16,32]. To adjust for the differences in climatological profile between the two countries, we shifted the dynamics fitted to the Tanzania data by a time lag equal to the difference between the starting times of the dry season in the two countries. Specifically, we defined this time lag by comparing historical rainfall averages for the time period 1901–2015 (electronic supplementary material, figure S4). We then implemented known measurements of the LF prevalence in rodents captured in Nigeria in the dry and rainy seasons separately, and we accounted for a non-zero probability of vertical transmission of the virus and its antibodies, as previously observed in rodents (see [16] for details).

### (e) Association of seasonal LF dynamics and climatological variables

To test for the significance of a causal relationship between the observed LF incidence and climatological variables, we employed an empirical dynamic modelling that is based on the convergent cross-mapping skill [20,21]. This method has previously been used to demonstrate the causal link between influenza transmission risk and humidity or temperature. The LF incidence was tested, searching for the significance of the association with one of the climatological variables, i.e. time series versus seasonal surrogates. The threshold of acceptance was chosen to be 0.01.

## Supplementary Material

Technical Appendix

## Data accessibility

We provide all of the accumulated data as electronic supplementary material.
