# Supplementary material for: Quantifying the seasonal drivers of transmission for Lassa fever in Nigeria
Source: Philos Trans R Soc Lond B Biol Sci. 2019 May 6;374(1775):20180268. doi: 10.1098/rstb.2018.0268 (PMC6553602; doi:10.1098/rstb.2018.0268)
Supplement: Technical Appendix [file rstb20180268supp1.docx]

*Appendix for*

Quantifying the seasonal driver of transmission for
Lassa fever in Nigeria

Andrei R. Akhmetzhanov, Yusuke Asai, Hiroshi Nishiura

**Contests**

1. **Analysis of a nosocomial outbreak in Jos, Nigeria, in 1970**

*Reconstructing the timeline of the outbreak*

*Inference of model parameters using the Bayesian approach*

*MCMC iterations*

1. **Model-based inference using the human case (incidence) data**

*Maximum likelihood estimation*

*Fitting procedure using the Bayesian approach*

*MCMC iterations*

1. **Dynamic modelling of LF transmission in rodent populations**

*Model formulation*

*Periodic conditions for the change in population size*

*Susceptible-infected-recovered (SIR) model*

*Inference of model parameters*

1. **Computer simulations and code sharing**

Appendix references

Appendix figures

Appendix code snippets

**A. Analysis of a nosocomial outbreak in Jos, Nigeria, in 1970**

*Reconstructing the timeline of the outbreak*

We used the available event time data of a nosocomial outbreak in Jos, Nigeria, in 1970, to estimate the incubation period and the time from illness onset to death. In total, the outbreak involved 23 cases excluding the index case. Each case record $j=\{1\ldots23\}$ contained a lower $t_{j}^{e-}$ and an upper time boundary $t_{j}^{e+}$ of the probable date of exposure to Lassa fever (LF) virus as well as two other event times, i.e., the date of illness onset $t_{j}^{o}$ and the date of death $t_{j}^{d}$ (Appendix Fig 1).

*Inference of model parameters using the Bayesian approach*

In our Markov Chain Monte Carlo (MCMC) iterations, we employed a normal distribution for the prior distribution of each exposure time, $t_{j}^{e}$:

$$t_{j}^{e} \sim\mathrm{dnorm}\left( \mathrm{mean}=(t_{j}^{e+}+t_{j}^{e-})/2, \mathrm{sd}=(t_{j}^{e+}-t_{j}^{e-})/2/1.96 \right).$$

The mean and standard deviation (sd) were used to ensure that the majority of values belonged to the interval between $t_{j}^{e-}$ and $t_{j}^{e+}.$ The normal distribution was preferred to the uniform prior $\mathrm{dunif}\left( \mathrm{lower}=t_{j}^{e-},\mathrm{upper}=t_{j}^{e+} \right)$ to avoid the edge effects from the boundaries [1], see also discussion in [2].

Then we defined two main distributions for the incubation period and the time from illness onset to death, and employed the gamma distribution for each:

$$t_{j}^{i} \sim\mathrm{dgamma}\left( \mathrm{shape}=\left( \frac{m_{i}}{{sd}_{i}} \right)^{2},\mathrm{rate}=\frac{m_{i}}{\left( sd_{i} \right)^{2}} \right),$$

$$t_{j}^{od} \sim\mathrm{dgamma}\left( \mathrm{shape}=\left( \frac{m_{d}}{sd_{d}} \right)^{2},\mathrm{rate}=\frac{m_{d}}{\left( sd_{d} \right)^{2}} \right),$$

where hyper-parameters, the means $m_{i}$ and $m_{d}$, and standard deviations ${sd}_{i}$ and ${sd}_{d}$, have non-informative positively defined priors given by a half flat (positive) distribution with arbitrarily small shape and scale parameters:

$$m_{i},m_{d},{sd}_{i},{sd}_{d} \sim dhalfflat( )$$

The observed times of illness onset $t_{j}^{o}$ and death $t_{j}^{d}$ were subsequently inferred using sampling from normal distributions with a fixed standard deviation 0.5, which correlates with a half of the time scale in the timeline as a source of measurement error:

$$t_{j}^{o} \sim\mathrm{dnorm}\left( \mathrm{mean}=t_{j}^{e}+t_{j}^{i}, \mathrm{sd}=0.5 \right),$$

$$t_{j}^{d} \sim\mathrm{dnorm}\left( \mathrm{mean}=t_{j}^{e}+t_{j}^{i}+t_{j}^{od}, \mathrm{sd}=0.5 \right).$$

*MCMC iterations*

We performed MCMC iterations with 500,000 iterations, plus 20,000 iterations as a burn-in period. The thinning parameter of $m_{i}, m_{d},sd_{i},$ and ${sd}_{d}$ was equal to 50, and the exposure time $t_{j}^{e}$ was set at 10. The resulting trace plots for the mean and variance complemented with autocorrelation plots shown in Appendix Figures 2–5 demonstrate the sufficient convergence power of the iterative process.

**B. Model-based inference using the human case (incidence) data**

*Maximum likelihood estimation*

As stated in the main text, we used maximum likelihood estimation (MLE) to obtain point estimates of model parameters $\boldsymbol{\theta=}\left\{ a_{\pm},q,l_{1,2} \right\}.$ Specifically, we maximized the total (composite) likelihood of the form:

$$L_{\Sigma}\left( \boldsymbol{\theta} | \left\{ i_{w}+d_{w},d_{w} \right\} \right)=L_{i}\left( \boldsymbol{\theta} | \{i_{w}+d_{w}\} \right) L_{d}\left( \boldsymbol{\theta} | \{i_{w}+d_{w},d_{w}\} \right),$$

with respect to three varied parameters $\{a_{\pm},q\}$ for a fixed pair of indices $l_{1,2}$. Then we performed a grid search over all possible values $l_{1,2},$ spanning the range $\left\{ 1,2,\ldots,52 \right\}$ to determine the global maximum of the likelihood $L_{\Sigma}.$ Each point estimate can be complimented with 95% confidence intervals, e.g., based on the likelihood profile.

Appendix Figure 6 shows the resulting fit using the MLE procedure. For comparison, the fit of the analogous model with a (single) constant exposure rate is shown in Appendix Figure 7. The single exposure rate model yielded a greater Akaike information criterion value (3679.2 in contrast to 2877.2 with two exposure model).

*Fitting procedure using the Bayesian approach*

We found the level of uncertainty in each model parameter estimate by conducting MCMC simulations for Bayesian inference. Here, we describe the sampling procedure for each estimate by inferring an underlying posterior distribution. Specifically, we were interested in estimating the posterior of the exposure rate *a* for each high/low-risk period, and the CFR *q*.

The exposure rate *a_w_* at week number *w* was sampled from the Gamma distribution:

$$a_{w}\left( l_{1,2} \right) \sim\mathrm{dgamma}\left( \mathrm{shape}=\left( \frac{m^{a}\left( w_{c}\left( w \right),l_{1,2} \right)}{{sd}^{a}\left( w_{c}\left( w \right),l_{1,2} \right)} \right)^{2}, \mathrm{scale}=\frac{m^{a}\left( w_{c}\left( w \right),l_{1,2} \right)}{\left( {sd}^{a}\left( w_{c}\left( w \right),l_{1,2} \right) \right)^{2}} \right),$$

where $w_{c}$ is a corresponding calendar week, whereas a pair of hyper-parameters $m^{a}$ and $sd^{a}$ are given by the following expressions:

$$m^{a}\left( w_{c},l_{1,2} \right)=\left\{ \begin{matrix} m_{1}^{a}, \mathrm{if} l_{1}\leq w_{c}\leq l_{2}, \\ m_{2}^{a}, \mathrm{otherwise}, \end{matrix} \right.$$

$${sd}^{a}\left( w_{c},l_{1,2} \right)=\left\{ \begin{matrix} {sd}_{1}^{a}, \mathrm{if} l_{1}\leq w_{c}\leq l_{2}, \\ {sd}_{2}^{a}, \mathrm{otherwise}. \end{matrix} \right.$$

We supplemented each with the following non-informative prior distributions: $m_{1,2}^{a},{sd}_{1,2}^{a} \sim dhalfflat( ),$ that are half flat (positive) distributions. The time boundaries $l_{1,2}$ had the uniform prior distributions: $l_{1,2} \sim\mathrm{dunif}\left( \mathrm{lower}=0,\mathrm{upper}=53 \right),$ which ensure: $0<l_{1,2}<53.$ We also imposed constraints: $m_{1}^{a}<m_{2}^{a}$ and $l_{1}+3<l_{2}.$

The risk of death *q* is sampled from a Gamma distribution to ensure the positive range of its values:

$$q \sim\mathrm{dgamma}\left( \mathrm{shape}=\left( \frac{m^{q}}{sd^{q}} \right)^{2}, \mathrm{scale}=\frac{m^{q}}{\left( sd^{q} \right)^{2}} \right),$$

where two hyper-parameters have the non-informative prior distributions of the form: $m^{q},sd^{q} \sim dhalfflat( ),$ that are analogous to the priors for the exposure hyper-parameters used above.

To infer parameters, we sampled the number of cases and the number of deaths from Poisson and Binomial distributions, respectively:

$$i_{w}+d_{w} \sim\mathrm{dpois}\left( \mathrm{rate}=\lambda_{w}^{i}+\lambda_{w}^{d} \right),$$

$$d_{w} \sim\mathrm{dbinom}\left( size=i_{w}+d_{w},\mathrm{prob}=\frac{\lambda_{w}^{d}}{\lambda_{w}^{i}+\lambda_{w}^{d}} \right),$$

where the rates $\lambda_{w}^{i}$ and $\lambda_{w}^{d}$ resulted from convolution sums. The first being defined by the formula:

$$\lambda_{w}^{i}=\sum_{k<w} \left( 1-q \right)\cdot\frac{a_{w-k}\times f_{k}}{1-r},$$

where $f_{w}$ denotes the incubation time distribution, see Appendix A. Whereas, the second rate was given by the following formula:

$$\lambda_{w}^{d}=\sum_{k<w} q\cdot\frac{a_{w-k}\times g_{k}}{1-r},$$

where $g_{w}$ is the distribution of time periods from exposure to death. In turn, we convoluted the distribution $f_{w}$ with the distribution of time periods from illness onset to death $h_{w}$ obtained earlier in Appendix A, i.e.:

$$g_{w}=\sum_{k<w} f_{w-k}\times h_{k}.$$

*MCMC iterations*

To obtain posterior distributions of model parameters $m_{1,2}^{a}, {sd}_{1,2}^{a}, m^{q}, {sd}^{q}, l_{1,2},$we compiled MCMC iterations with 18 chains, each characterized by 100,000 iterations and a burn-in period consisting of 20,000 iterations (Appendix Code Snippets). The thinning parameter for all parameters was set to 100 to avoid correlation effects in the chain. The resulting trace and density plots, as well as the autocorrelation plots for each pair of parameters, are shown in Appendix Figures 8–16. As shown, a sufficient level of convergence of the iterative algorithm was obtained.

**C. Dynamic modelling of LF transmission in rodent populations**

*Model formulation*

To model the transmission dynamics of LF in rodents, we adopted the modelling framework reported by Peel *et al.* [3]. We let $N\left( t \right)$ be the population size of rodents at time *t* ($0\leq t<1$ – where the season length is scaled to one). Then, the dynamics could be represented as follows:

$$\frac{dN\left( t \right)}{dt}=\left( B_{0}\left( t \right)-\mu\right)N\left( t \right),$$

$$N\left( t \right)=N\left( t+1 \right), 0\leq t<1,$$

where $\mu$ is the mortality rate *per capita*, and the growth rate *per capita* $B_{0}\left( t \right)$ is given by a periodic Gaussian function: $B_{0}\left( t \right)=\kappa\exp\left( -s\cos^{2} \left( \pi t-\phi\right) \right).$ The mortality rate for rodent species *Mastomys natalensis* is remarkably high: $\mu=7 \mathrm{year}^{-1},$ whereas the two parameters $s$ and $\phi$ were previously identified from 20-year observations of rodent species in Tanzania: $s=2.7,$ $\phi=0.4,$ see [4] and Supplementary Figure 1 therein. To adjust the model for a situation in Nigeria, we considered a lower average population density of 60 rodents per Ha compared to 80 rodents per Ha as in [4]. Whereas the seasonality parameter $s$ will be the subject of the model fit.

Due to apparent differences in climate between Tanzania and Nigeria, such as changes in the timing and length of the rainy season, a time shift was imposed to the function $B_{0}\left( t \right)$ to obtain the seasonal population dynamics in Nigeria. Specifically, we first identified the end of the rainy season in both countries based on historical averages of rainfall over the time period 1901–2015: $t_{TZ}=0.35$ for part of the year for Tanzania, $t_{NG}=0.83$ for part of the year for Nigeria (Appendix Figure 16). Then we calculated the difference between these two times as $\tau=t_{NG}-t_{TZ}=0.48$ for part of the year, and accounted for the shift in the birth rate function of this value: $B\left( t \right)=B_{0}(t-\tau)$ (Fig S3*b*).

*Periodic conditions for the change in population size*

We chose a parameter *κ* to satisfy the periodicity condition for $N(t).$ We required: $N\left( 0 \right)=N\left( 1 \right),$ which translates to:

$$\ln\frac{N\left( 1 \right)}{N(0)}=\int_{0}^{1} \left( B\left( t \right)-\mu\right) dt=\int_{0}^{1} \kappa e^{-s\cos^{2} (\pi t-\phi)} dt-\mu=0.$$

This rewrites as follows:

$\kappa\int_{0}^{1} \exp\left( -s\frac{\cos\left( 2\left( \pi t-\phi\right) \right)+1}{2} \right) dt-\mu=\frac{\kappa e^{-\frac{s}{2}}}{2 \pi} \int_{0}^{1} \exp\left( -\frac{s}{2}\cos\left( 2\pi t-2\phi\right) \right) d\left( 2\pi t-2\phi\right)-\mu=\frac{\kappa e^{-\frac{s}{2}}}{2 \pi} \int_{0}^{2\pi} \exp\left( -\frac{s}{2}\cos\psi\right) d\psi-\mu=\frac{\kappa e^{-\frac{s}{2}}}{\pi} \int_{0}^{\pi} \exp\left( -\frac{s}{2}\cos\psi\right) d\psi-\mu=\frac{\kappa e^{-\frac{s}{2}}}{\pi} I_{0}\left( s/2 \right)-\mu=0,$

to obtain: $\kappa=\frac{\mu e^{s/2}}{I_{0}\left( s/2 \right)}.$ Here, $I_{0}\left( x \right)=\pi^{-1}\int_{0}^{\pi} \exp\left( x\cdot\cos\left( \varphi\right) \right)d\varphi$ denotes the Bessel function of the first kind. Thus, we did not need to assign an estimate for parameter $\kappa$.

We also note that the average population density can be defined as follows:

$$\bar{N}=N\left( 0 \right)\int_{0}^{1} \exp\left( \int_{0}^{t} \left( B\left( \tau\right)-\mu\right) d\tau\right) dt.$$

*Susceptible-infected-recovered (SIR) model*

The flow diagram (Fig S3*a*) translates into the following dynamical equations:

$$\frac{dS\left( t \right)}{dt}=B\left( t \right)\left[ S\left( t \right)+\left( 1-\varepsilon\right)I\left( t \right)+\left( 1-\omega\right)R\left( t \right) \right]-\frac{\beta S\left( t \right)I\left( t \right)}{N\left( t \right)}+\lambda R\left( t \right)-\mu S\left( t \right),$$

$$\frac{dI\left( t \right)}{dt}=\varepsilon B\left( t \right)I\left( t \right)+\frac{\beta S\left( t \right)I\left( t \right)}{N\left( t \right)}-\left( \gamma+\mu\right)I\left( t \right),$$

$$\frac{dR\left( t \right)}{dt}=\omega B\left( t \right)R\left( t \right)+\gamma I\left( t \right)-\left( \lambda+\mu\right)R\left( t \right),$$

for the three components: susceptible $S\left( t \right),$ infected $I\left( t \right),$ and recovered $R\left( t \right),$ respectively. All rates are defined as *per capita*, $\gamma$ and $\lambda$ denote transition rates between compartments $S$ and $I,$ and $I$ and $R,$ respectively. Two other rates $\varepsilon$ and $\omega$ are the rates of vertical (parental) transmission of the LF virus and its antibodies, respectively. The transmission coefficient is set to a variable $\beta=v\cdot c$, which consists of the rate $w$ at which a susceptible rodent becomes infectious when in contact with an infected rodent, and contact-density function $c=c\left( N \right).$ The latter has been previously found by fitting the observed experimental data of contact pattern in rodents to the sigmoidal function [4,5]:

$$c\left( N \right)=\frac{2.13}{1+\exp(-0.05\left( N-101.2 \right))}.$$

We translated the absolute quantities of infected and recovered rodents to their densities with respect to the total size of the population. We also introduced two new variables: $x\left( t \right)=I(t)/N(t),$ and $z\left( t \right)=R(t)/N(t)$, and redefined the system of dynamic equations written above as follows:

|  | $\frac{dN\left( t \right)}{dt}=\left( B\left( t \right)-\mu\right)N\left( t \right),$  $\frac{dx\left( t \right)}{dt}=vc\left( N\left( t \right) \right)x\left( t \right)\left( 1-x\left( t \right)-z\left( t \right) \right)-\left( \left( 1-\varepsilon\right)B\left( t \right)+\gamma\right)x\left( t \right),$  $\frac{dz\left( t \right)}{dt}=\gamma x\left( t \right)-\left( \left( 1-\omega\right)B\left( t \right)+\lambda\right)z\left( t \right),$ | (S1) |
| --- | --- | --- |

The number of susceptible rodents is given by: $S\left( t \right)=N\left( t \right)\left( 1-x\left( t \right)-z\left( t \right) \right).$

*Inference of model parameters*

To predict the transmission dynamics of LF infection among rodents, we use documented facts on the course of infection [6]: (i) LF infection is asymptomatic in rodents; (ii) it is also transient, with the virus being cleared from the blood after the infection; (iii) LF virus can be secreted in urine up to 103 days; (iv) there is a probability of horizontal and vertical transmission of LF virus and its antibodies. This results in the following choice of parameters, see Fig S3*c*. In particular, we define the characteristic recovery time from the infection as 90 days, and the rate of recovery is therefore set to: $1/{90} \mathrm{days}^{-1}=4.06 \mathrm{year}^{-1}.$ The characteristic time at which immunity is lost is assigned as 120 days, i.e., the respective rate is set to: $1/{120} \mathrm{days}^{-1}=3.04 \mathrm{year}^{-1}.$

However, we still do not know the rate $v$ between susceptible and infected rodents that characterizes the transmission dynamics in the SIR model, the seasonality parameter $s$ and possibly unknown average population size $\bar{N}.$ Identification of this becomes a subject of the model fit.

To determine the values of model parameters, we required the mean prevalence levels of LF infection among rodents in the dry and rainy seasons, defined as $\bar{x}_{\mathrm{dry}}$ and $\bar{x}_{\mathrm{rny}},$ to be close to the observed levels in the field experiments. Fichet–Calvet and colleagues [6] reported that a one-time measurement in the dry season revealed the mean prevalence to be at 29%, while five independent measurements (one in the dry season and four in the rainy season) showed the mean level of prevalence to be 43%. Hence, we adjusted the mean prevalence level separately for both the dry and rainy seasons by using a simple algebraic rule: if $x_{\mathrm{rny}}$ is the mean prevalence level observed in the rainy season, then it should satisfy the following condition for five independent measurements: $1/5\times29\%+4/5\times x_{\mathrm{rny}}=43\%.$ This yields the value: $x_{\mathrm{rny}}=46.5\%.$ Thus, we required the values $\bar{x}_{\mathrm{dry}}$ and $\bar{x}_{\mathrm{rny}}$ predicted by our model to be close to 29% and 46.5%, respectively.

A fitting procedure was carried out by using the *χ*^2^ statistic. Specifically, we tried to minimize the quantity:

$$\chi^{2}=\frac{\left( 29\%-\bar{x}_{\mathrm{dry}} \right)^{2}}{\bar{x}_{\mathrm{dry}}}+\frac{\left( 46.5\%-\bar{x}_{\mathrm{rny}} \right)^{2}}{x_{\mathrm{rny}}},$$

where $\bar{x}_{\mathrm{dry}}$ and $\bar{x}_{\mathrm{rny}}$ are values obtained from the dynamics (S1). Appendix Figure 18 shows that the minimum of $\chi^{2}$ is reached on some one-dimensional manifold in the space of two parameters $\bar{N}$ and $s.$ The fitted dynamics and the evaluated values of $v$and $s$ for fixed $\bar{N}=60 \mathrm{Ha}^{-1}$ are shown in Figure S3.

**D. Computer simulations and code sharing**

All calculations were made using free, open-source statistical and programming environments (*R* Version 3.5.1, *Python* Version 3.6.6, and *Julia* Version 1.0.1). The results were tested in multiple computational environments to ensure the validity of the calculations and to avoid processing errors. MCMC simulations were performed using the *R* package NIMBLE Version 0.6-12 [7], and comparative analysis of different packages can be found elsewhere [8]. To run the significance test of association between the observed LF incidence and climatological variables, we used the *R* package rEDM [9]. The code for all calculations and to reproduce all of the figures are accessible from the open-shared repository: http://tiny.cc/Lassa18Scripts. This information can be used freely for non-commercial purposes.

Appendix References

1. Carpenter B. 2017 Computational and statistical issues with uniform interval priors. Available at: andrewgelman.com/2017/11/28/computational-statistical-issues-uniform-interval-priors/. Accessed on August 30, 2018.

2. Park SW, Dushoff J, Earn DJD, Poinar H, Bolker BM. 2018 Human ectoparasite transmission of the plague during the Second Pandemic is only weakly supported by proposed mathematical models. *Proc. Natl. Acad. Sci.* **115**, E7892–E7893. (doi:10.1073/pnas.1809775115)

3. Peel AJ, Pulliam JRC, Luis AD, Plowright RK, O’Shea TJ, Hayman DTS, Wood JLN, Webb CT, Restif O. 2014 The effect of seasonal birth pulses on pathogen persistence in wild mammal populations. *Proc. R. Soc. B Biol. Sci.* **281**, 20132962–20132962. (doi:10.1098/rspb.2013.2962)

4. Borremans B, Reijniers J, Hens N, Leirs H. 2017 The shape of the contact–density function matters when modelling parasite transmission in fluctuating populations. *R. Soc. Open Sci.* **4**, 171308. (doi:10.1098/rsos.171308)

5. Borremans B, Reijniers J, Hughes NK, Godfrey SS, Gryseels S, Makundi RH, Leirs H. 2017 Nonlinear scaling of foraging contacts with rodent population density. *Oikos* **126**, 792–800. (doi:10.1111/oik.03623)

6. Fichet-Calvet E, Becker-Ziaja B, Koivogui L, Günther S. 2014 Lassa Serology in Natural Populations of Rodents and Horizontal Transmission. *Vector-Borne Zoonotic Dis.* **14**, 665–674. (doi:10.1089/vbz.2013.1484)

7. de Valpine P, Turek D, Paciorek CJ, Anderson-Bergman C, Lang DT, Bodik R. 2017 Programming With Models: Writing Statistical Algorithms for General Model Structures With NIMBLE. *J. Comput. Graph. Stat.* **26**, 403–413. (doi:10.1080/10618600.2016.1172487)

8. Li M, Dushoff J, Bolker BM. 2018 Fitting mechanistic epidemic models to data: A comparison of simple Markov chain Monte Carlo approaches. *Stat. Methods Med. Res.* **27**, 1956–1967. (doi:10.1177/0962280217747054)

9. Ye H, Clark A, Deyle E, Munch S, et al. In press. *rEDM: Applications of Empirical Dynamic Modeling from Time Series. R package version 0.7.1.*

10. Carey DE, Kemp GE, White HA, Pinneo L, Addy RF, Fom ALMD, Stroh G, Casals J, Henderson BE. 1972 Lassa fever Epidemiological aspects of the 1970 epidemic, Jos, Nigeria. *Trans. R. Soc. Trop. Med. Hyg.* **66**, 402–408. (doi:10.1016/0035-9203(72)90271-4)

11. Sluydts V, Davis S, Mercelis S, Leirs H. 2009 Comparison of multimammate mouse (Mastomys natalensis) demography in monoculture and mosaic agricultural habitat: Implications for pest management. *Crop Prot.* **28**, 647–654. (doi:10.1016/j.cropro.2009.03.018)

12. University of East Anglia Climatic Research Unit, Jones PD, Harris IC. 2008 Climatic Research Unit (CRU) time-series datasets of variations in climate with variations in other phenomena. NCAS British Atmospheric Data Centre. Assessed August 2017.

Appendix Figures

**Appendix Fig 1: Timeline of a nosocomial outbreak in Jos, Nigeria, in 1970, adapted from** [10]**.** Shaded grey areas indicate the exposure period, while shaded yellow areas span the time period from illness onset to death.


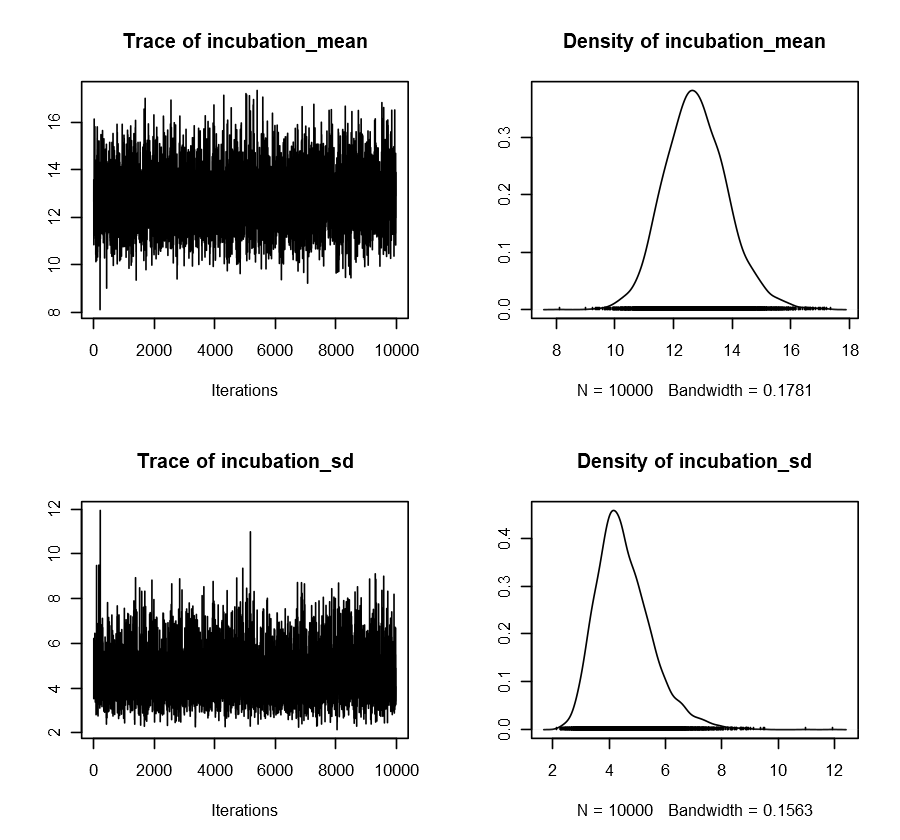


**Appendix Figure 2: Trace and density plots for the mean** $\boldsymbol{m}_{\boldsymbol{i}}$ **(“incubation_mean”) and standard deviation** $\boldsymbol{sd}_{\boldsymbol{i}}$ **(“incubation_sd”) of the incubation period distribution.**


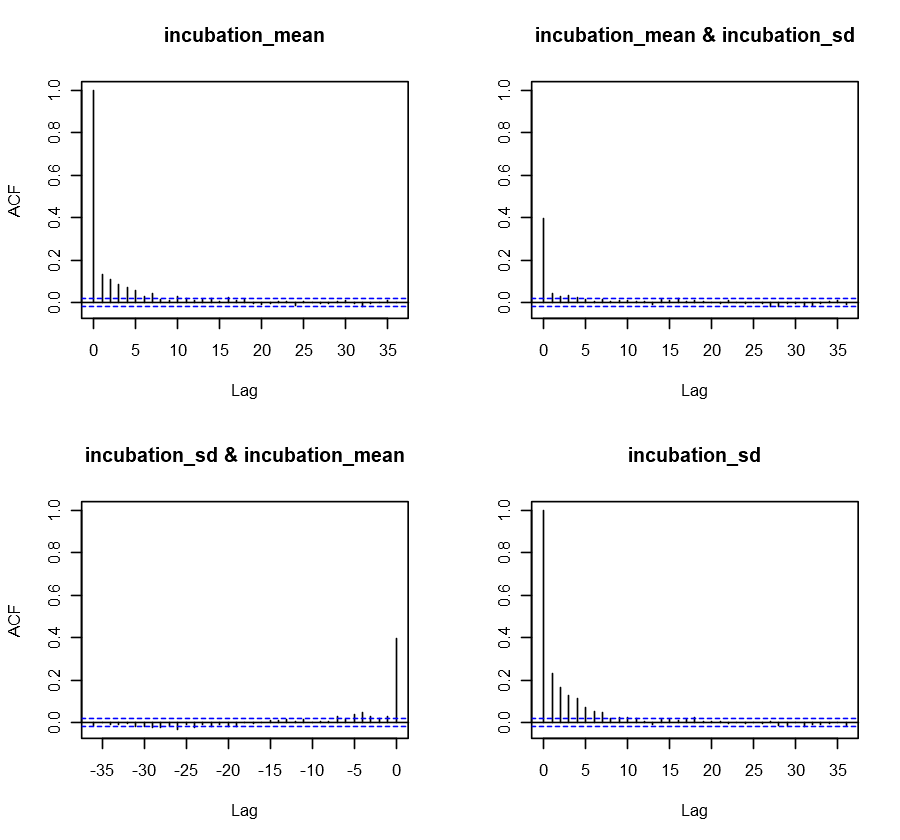


**Appendix Figure 3: Autocorrelation plots for pairs of mean** $\boldsymbol{m}_{\boldsymbol{i}}$ **(“incubation_mean”) and standard deviation** $\boldsymbol{sd}_{\boldsymbol{i}}$ **(“incubation_sd”) values for the incubation period distribution.**


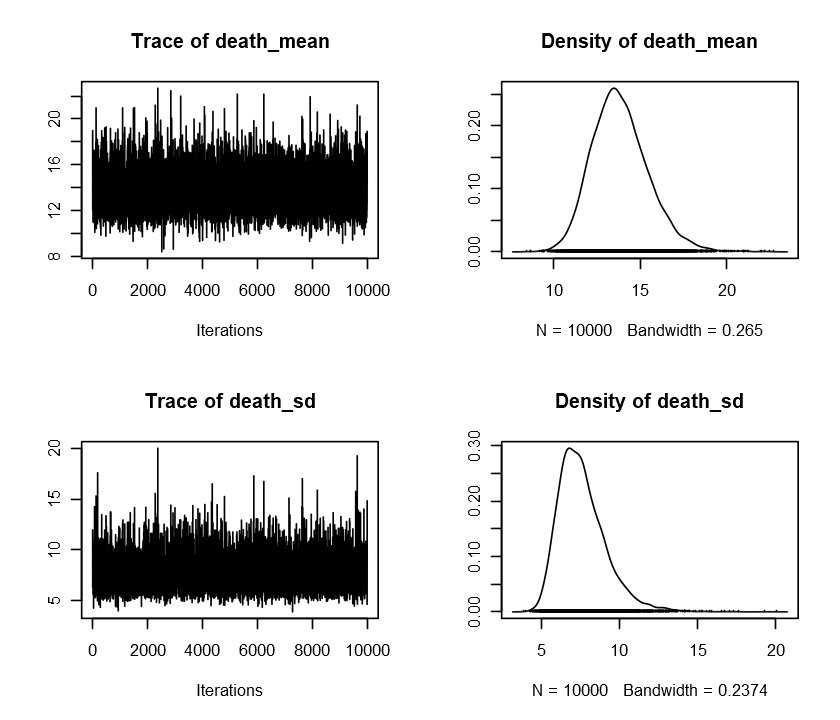


**Appendix Figure 4: Trace and density plots for the mean** $\boldsymbol{m}_{\boldsymbol{d}}$ **(“death_mean”) and standard deviation** $\boldsymbol{sd}_{\boldsymbol{d}}$ **(“death_var”) of the distribution of the time period between illness onset and death.**


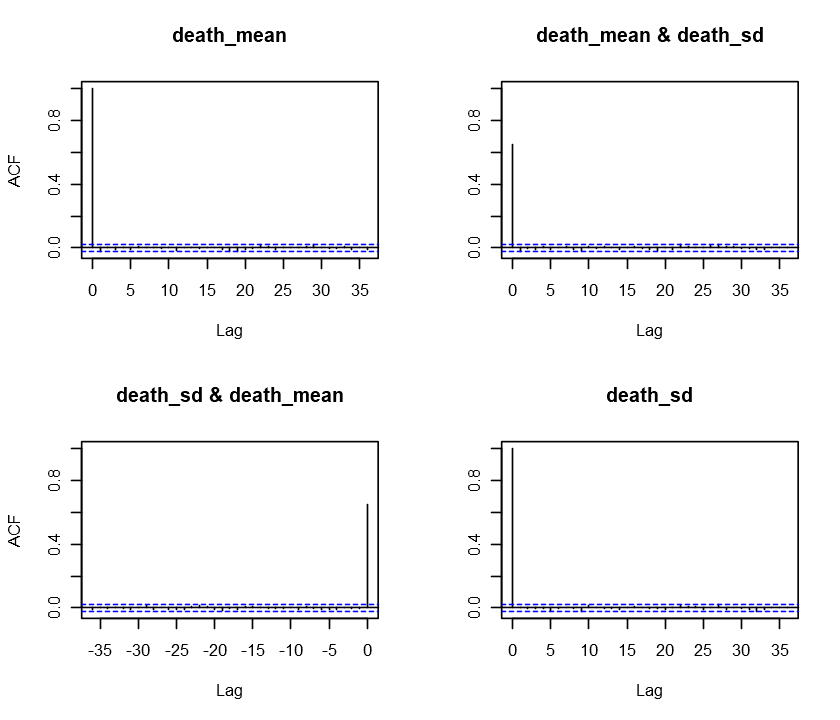


**Appendix Figure 5: Autocorrelation plots for pairs of mean** $\boldsymbol{m}_{\boldsymbol{d}}$ **(“death_mean”) and standard deviation** $\boldsymbol{sd}_{\boldsymbol{d}}$ **(“death_sd”) values for the distribution of the time period between illness onset and death.**

**
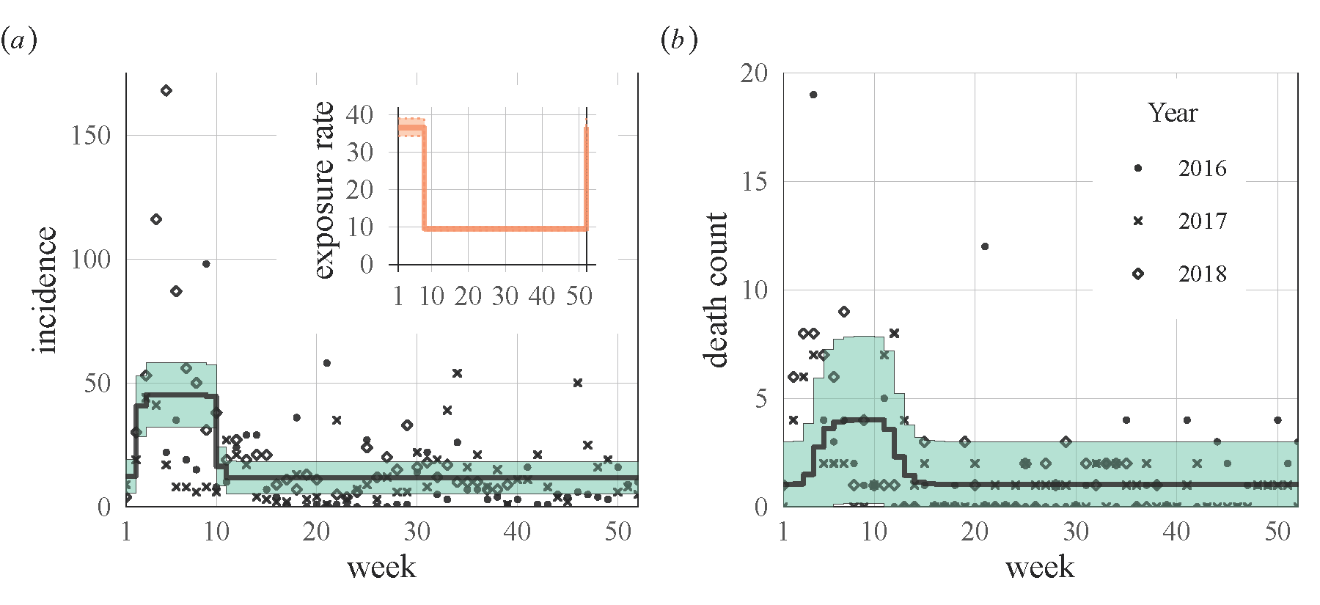
**

**Appendix Figure 6: Model fit to the observed data of new cases (*a*) and fatal cases (*b*) using maximum likelihood estimation.** Solid black line indicates the obtained average, whereas the shaded area shows the 95% profile-based confidence intervals. The fitted exposure rate as a function of calendar week is shown in the inset of (a).

**
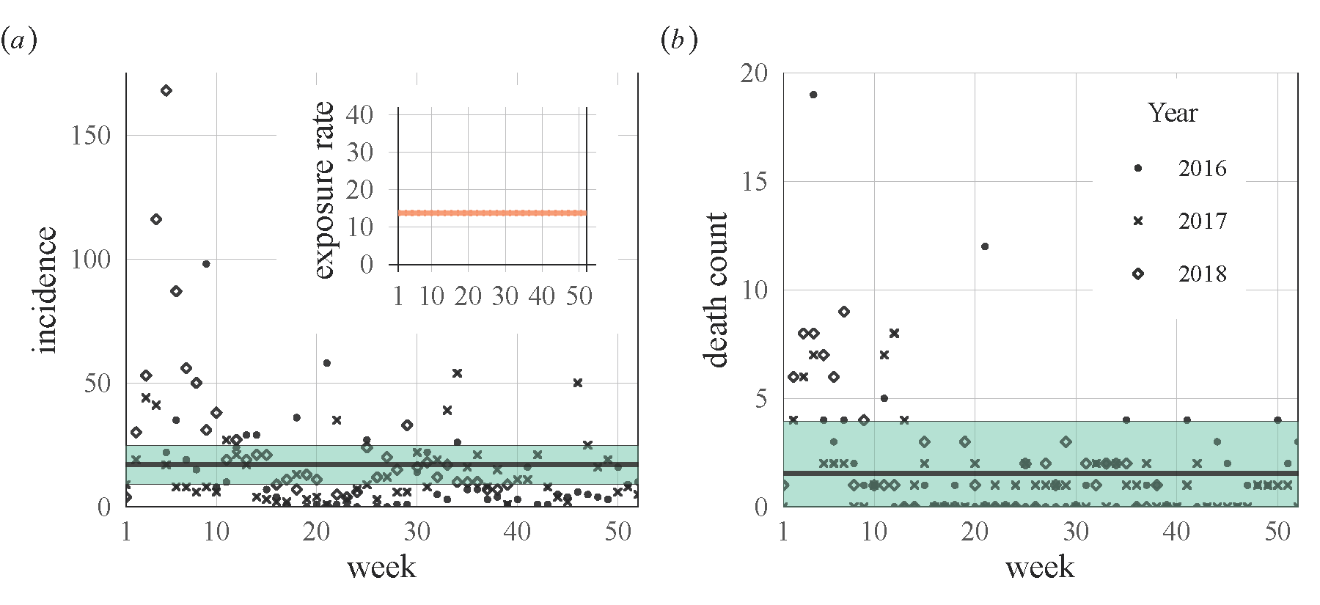
**

**Appendix Figure 7: Model fit to the observed data of new cases (*a*) and fatal cases (*b*) using maximum likelihood estimation for the alternative model with (single) constant exposure rate.** Solid black line indicates the obtained average, whereas the shaded area shows the 95% profile-based confidence intervals. The fitted exposure rate as a function of calendar week is shown in the inset of (a).


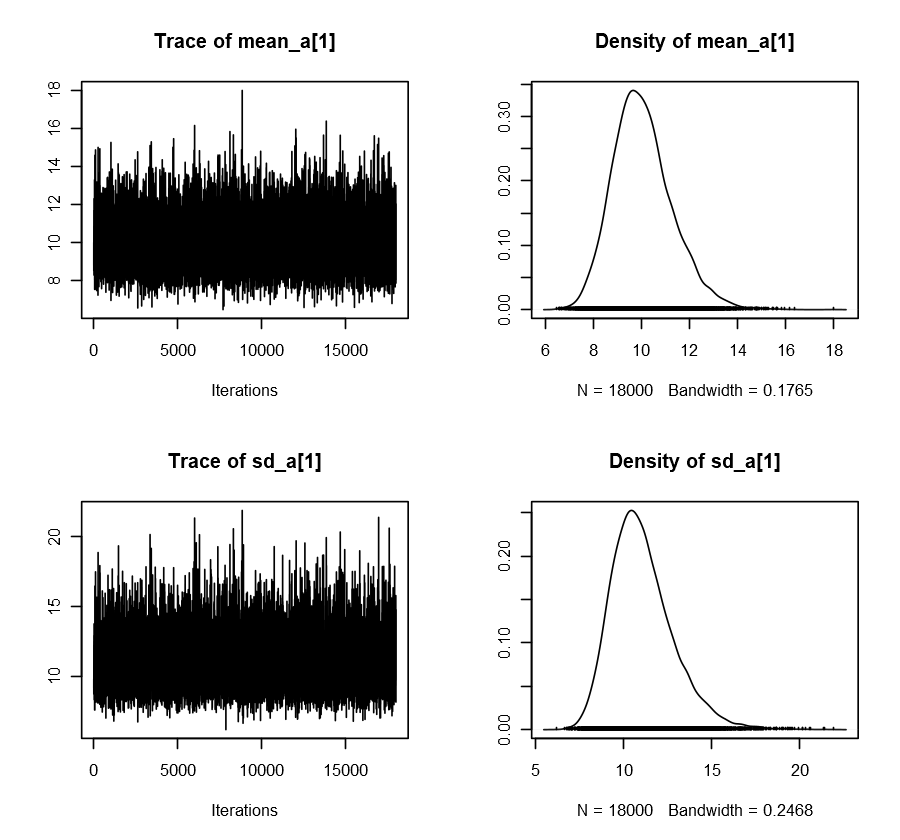


**Appendix Figure 8: Trace and density plots for** $\boldsymbol{m}_{\boldsymbol{1}}^{\boldsymbol{a}}$ **and** $\boldsymbol{sd}_{\boldsymbol{1}}^{\boldsymbol{a}}$**.**


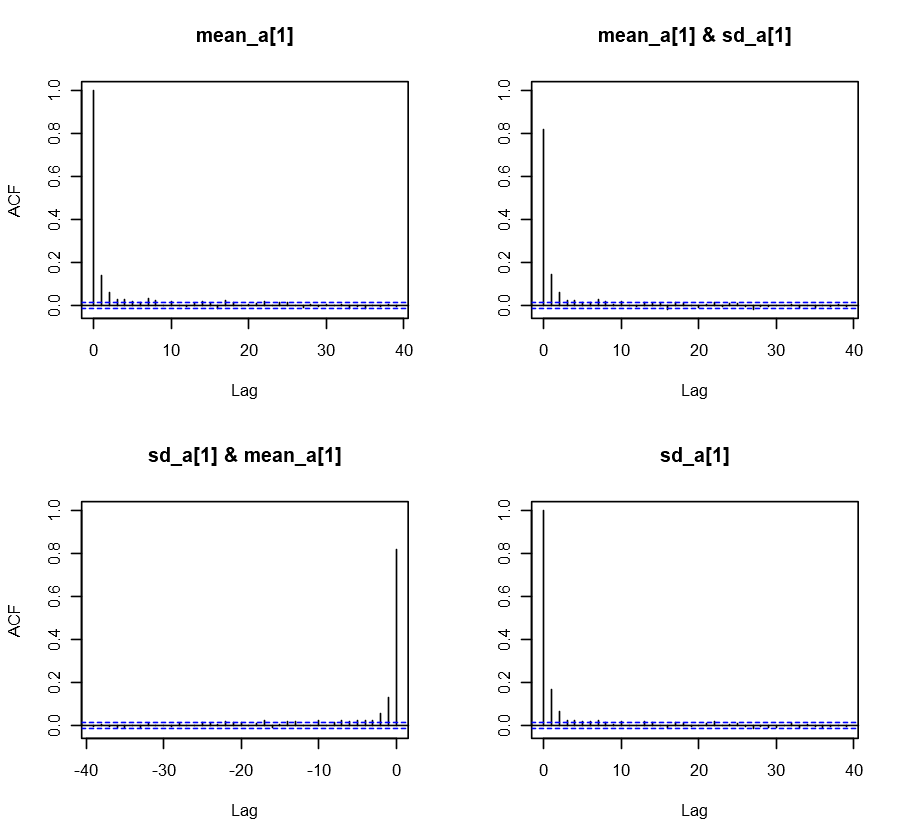


**Appendix Figure 9: Autocorrelation plots for the pair** $\boldsymbol{m}_{\boldsymbol{1}}^{\boldsymbol{a}}$ **and** $\boldsymbol{sd}_{\boldsymbol{1}}^{\boldsymbol{a}}$**.**


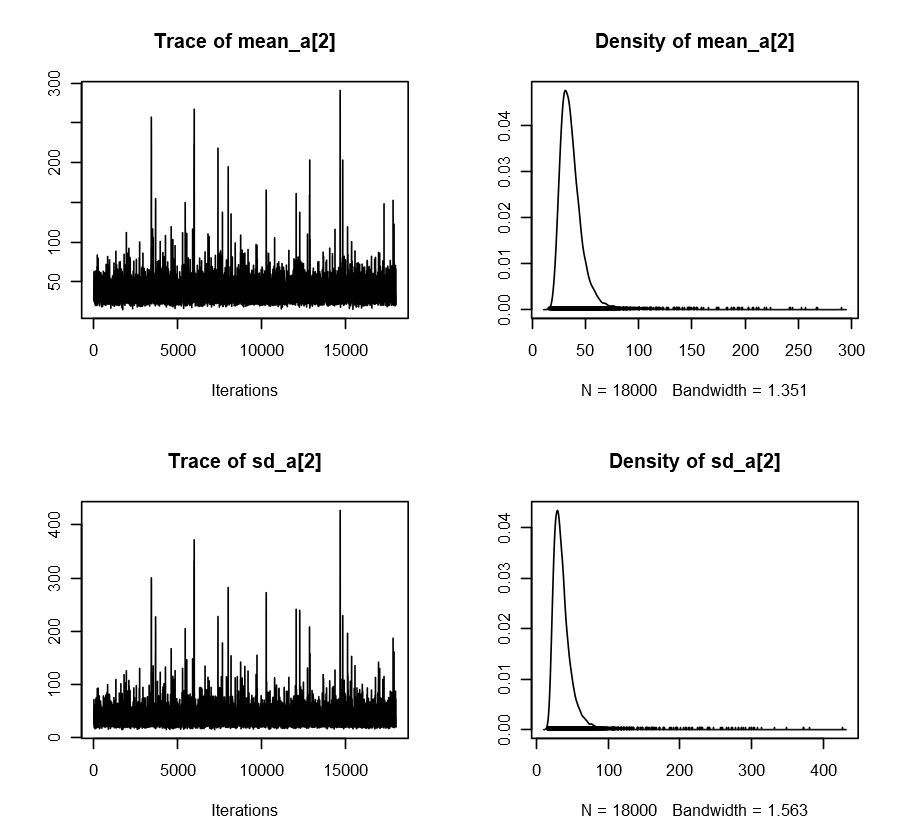


**Appendix Figure 10: Trace and density plots for** $\boldsymbol{m}_{\boldsymbol{2}}^{\boldsymbol{a}}$ **and** $\boldsymbol{sd}_{\boldsymbol{2}}^{\boldsymbol{a}}$**.**


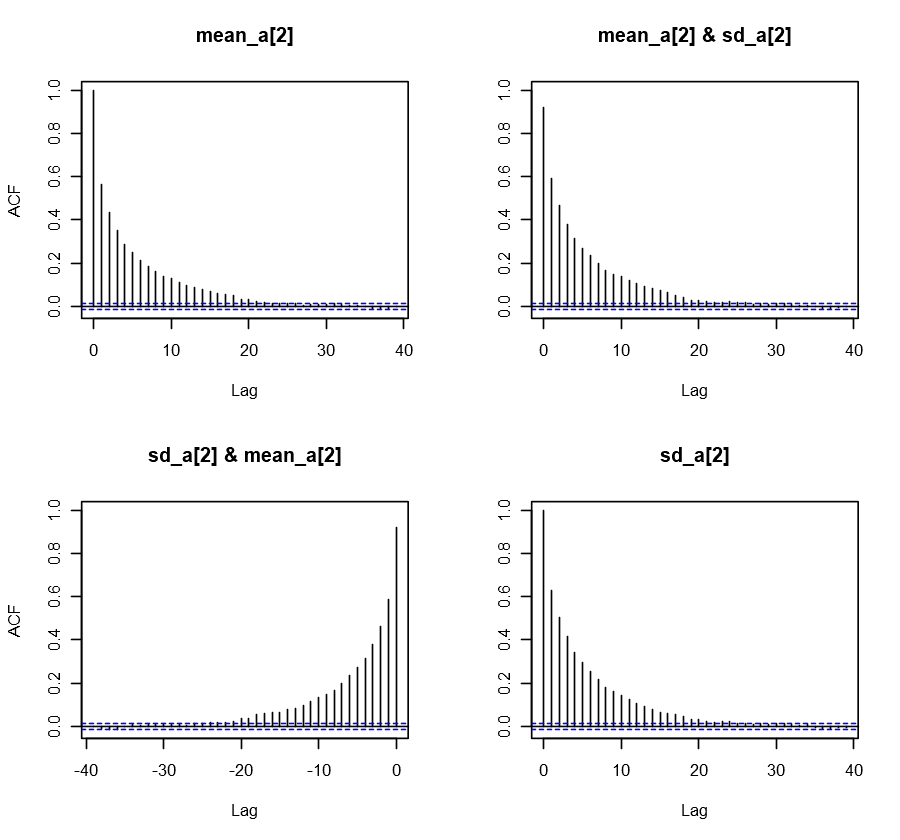
**Appendix Figure 11: Autocorrelation plots for the pair** $\boldsymbol{m}_{\boldsymbol{2}}^{\boldsymbol{a}}$ **and** $\boldsymbol{sd}_{\boldsymbol{2}}^{\boldsymbol{a}}$**.**


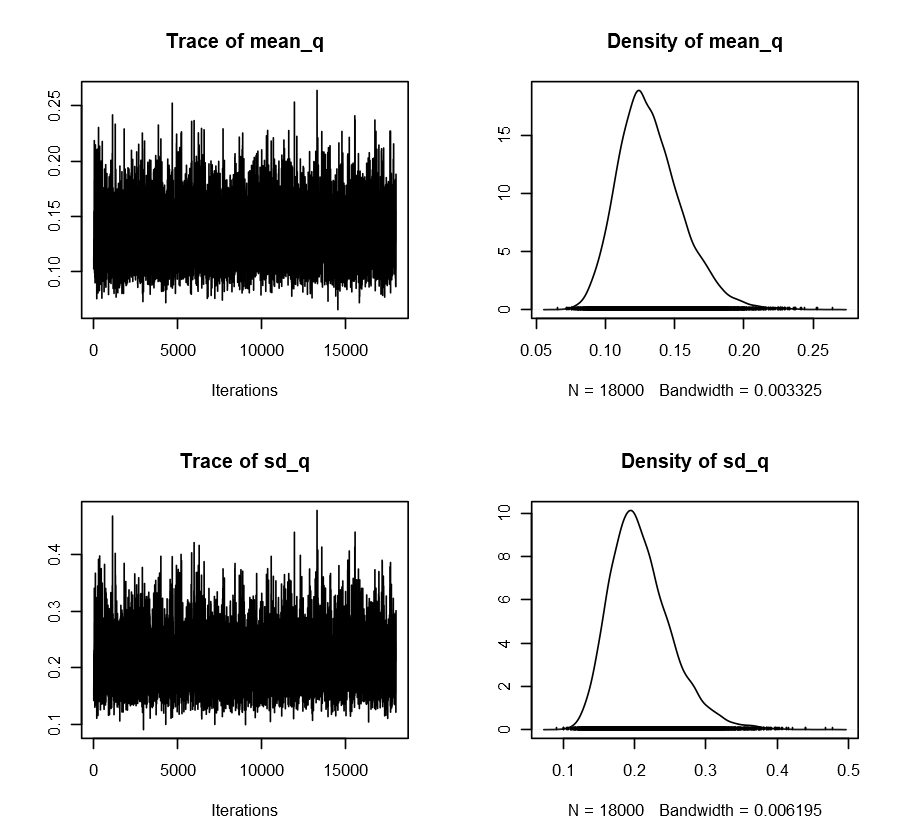


**Appendix Figure 12: Trace and density plots for** $\boldsymbol{m}^{\boldsymbol{q}}$ **and** $\boldsymbol{sd}^{\boldsymbol{q}}$**.**


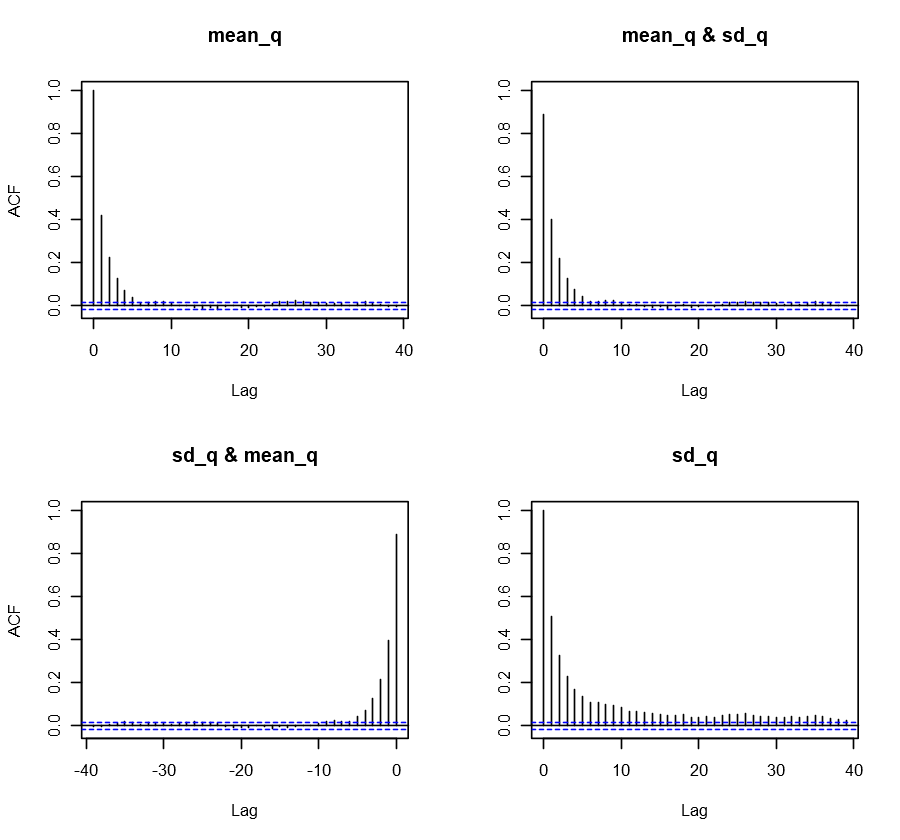


**Appendix Figure 13: Autocorrelation plots for the pair** $\boldsymbol{m}^{\boldsymbol{q}}$ **and** $\boldsymbol{sd}^{\boldsymbol{q}}$**.**


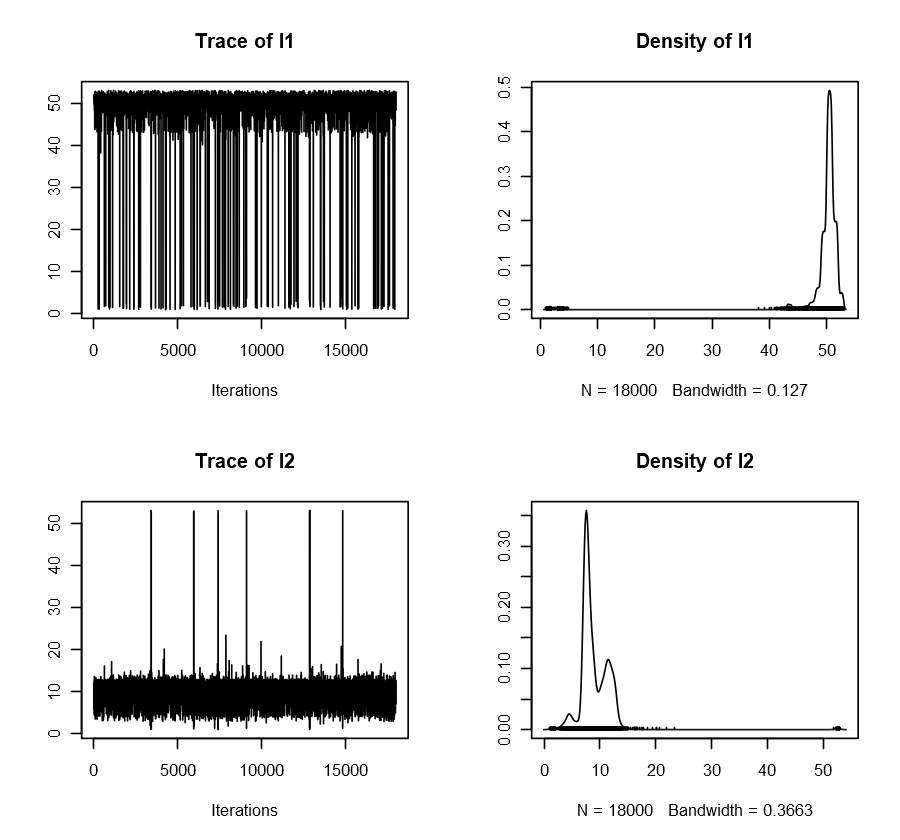
**Appendix Figure 14: Trace and density plots for** $\boldsymbol{l}_{\boldsymbol{1}}$ **and** $\boldsymbol{l}_{\boldsymbol{2}}$**.**


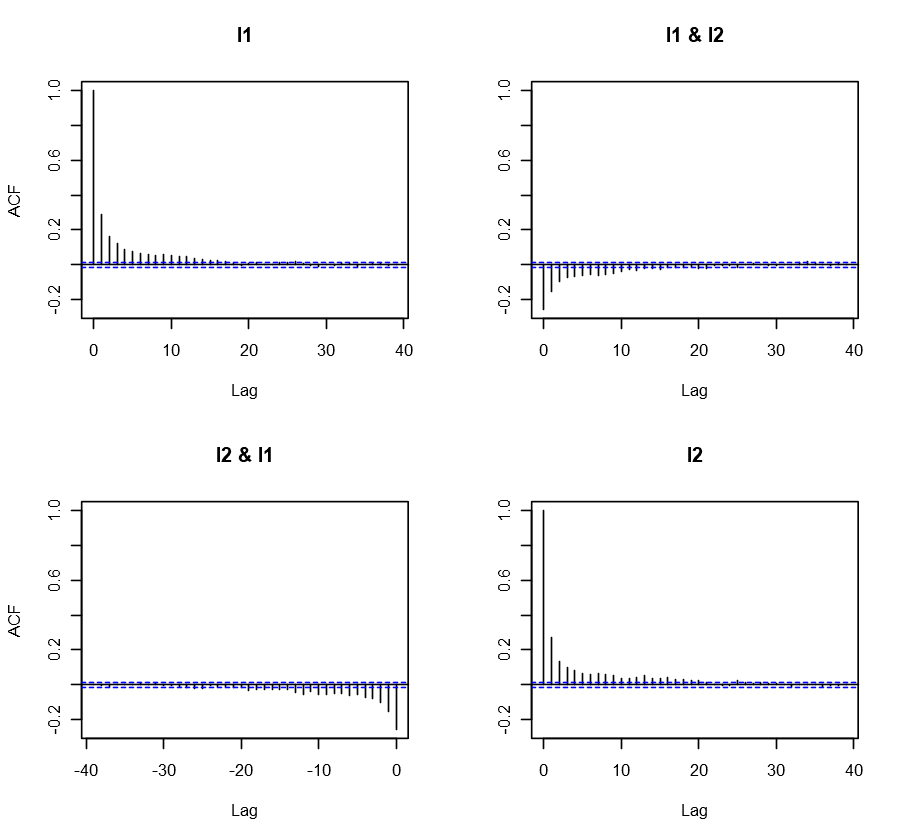


**Appendix Figure 15: Autocorrelation plots for the pair** $\boldsymbol{l}_{\boldsymbol{1}}$ **and** $\boldsymbol{l}_{\boldsymbol{2}}$**.**


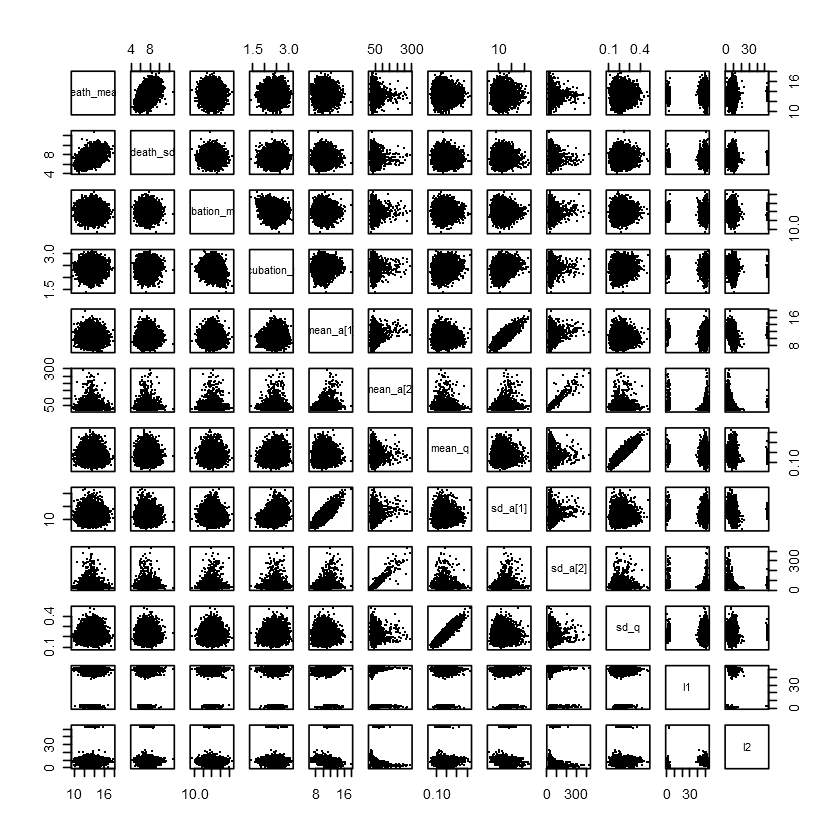


**Appendix Figure 16: Inference of the model parameters using MCMC iterations.**

**
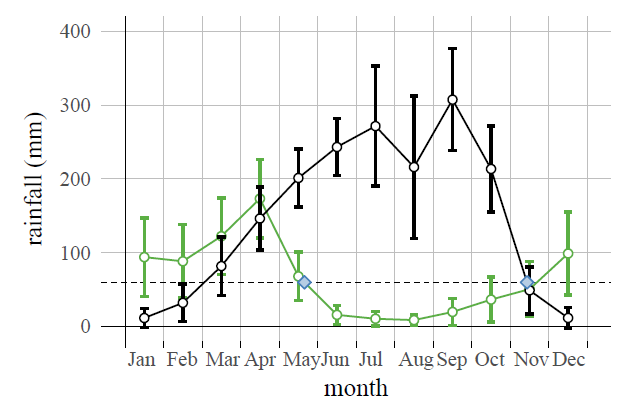
**

**Appendix Figure 17: Difference in rainfall patterns between Nigeria (black) and Tanzania (green).** The location used for Tanzania was $6^{^{\circ}}51'$S, $37^{^{\circ}}38'$E, which was the data collection site in the original study [11]. The closeness was restrained by gridded data point distribution in the global precipitation dataset [12]. Blue points indicate the starting points for the dry seasons.

**
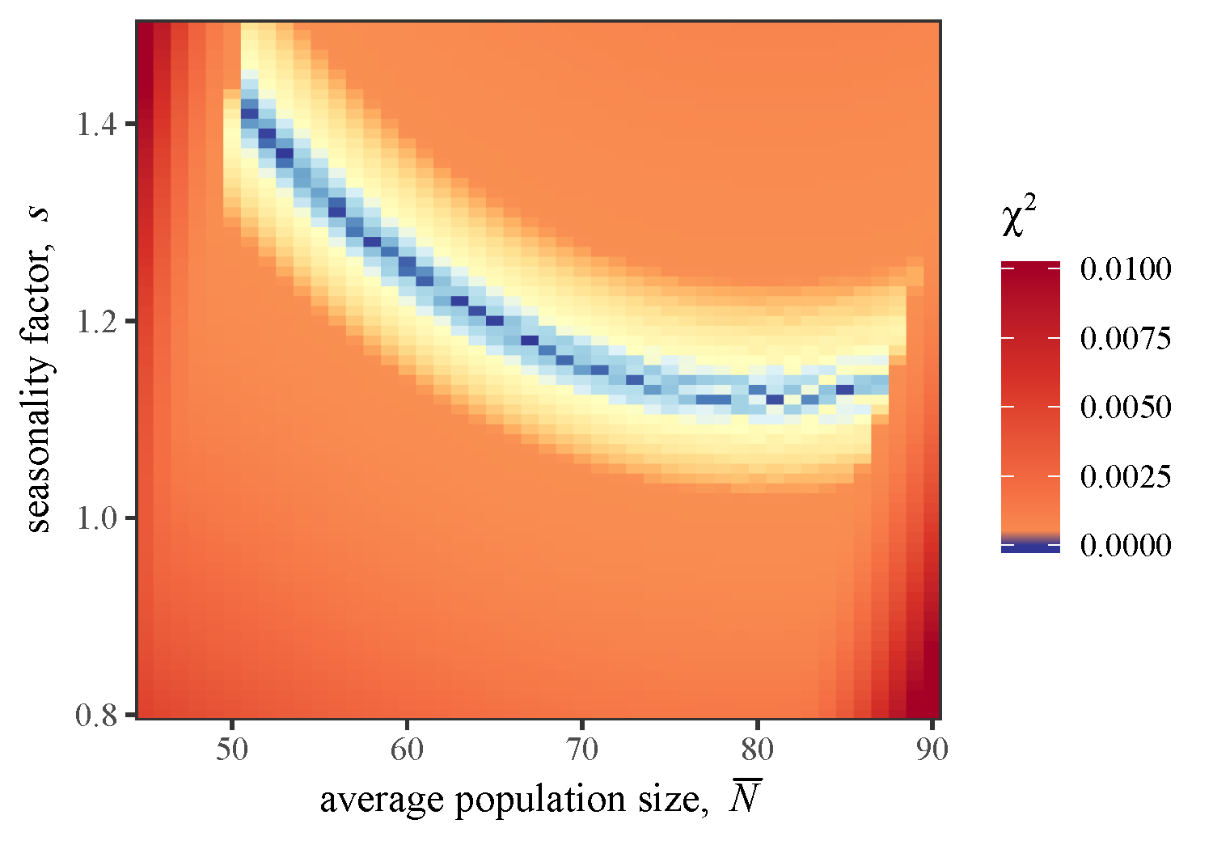
**

**Appendix Figure 18: Numerical minimization of the** $\boldsymbol{\chi}^{\boldsymbol{2}}$ **value over two parameters** $\boldsymbol{s}$ **and** $\bar{\boldsymbol{N}}$**.**

**Appendix Code Snippet 1: Main R script including the NIMBLE module**

## Preamble

args = commandArgs(trailingOnly=TRUE)

set.seed(as.numeric(args[1]))

libraries = c("dplyr","magrittr","tidyr","readxl","nimble")

for(x in libraries) { library(x,character.only=TRUE,warn.conflicts=FALSE) }

'%&%' = function(x,y)paste0(x,y)

rho = 0.19

## Data for to describe Jos outbreak

#### excluding index case

onsetTimes = c(17, 19, 21, 21, 21, 22, 22, 23, 23, 23, 23, 23, 25, 26, 26, 26, 27, 31, 39, 41, 41, 42, 44)

deathTimes = c(33, 48, 31, 47, 30, 41, 37, 32, 31, 35, 54, 30, 31, 33, 30, 33, 48, 39, 51, 48, 55, 60, 62)

## Prior knowledge

exposureTimesLower = c(5, 9, 5, 9, 5, 5, 5, 11, 5, 5, 13, 11, 12, 5, 11, 11, 5, 5, 12, 25, 5, 25, 25)

exposureTimesUpper = c(15, 18, 18, 15, 18, 18, 18, 15, 18, 18, 14, 15, 18, 18, 15, 15, 18, 18, 18, 31, 18, 31, 31)

## Dataset of human incidence stored in Nigeria_raw.xlsx

yearMin = 2016

data = read_excel("../../data/Nigeria_raw.xlsx", sheet = "Incidence") %>%

select(-one_of("Timeseries","Imputation","File in the repo"),-contains("URL")) %>%

filter(Year>=yearMin) %>%

group_by(Year) %>%

mutate(Incidence_Reported = if_else(Week==1,Reported,Reported-lead(Reported)),

Incidence_Deaths = if_else(Week==1,Deaths,Deaths-lead(Deaths))

) %>%

ungroup

data %>% tail

data %>% select(Year,Week,starts_with("Incidence")) -> Df

data.frame(Year=yearMin-1,Week=1:52,Incidence_Reported=NA,Incidence_Deaths=NA) %>%

rbind(Df %>% arrange(Year,Week)) %>%

rowwise %>%

mutate(Incidence_Reported_NA=ifelse(is.na(Incidence_Reported),rpois(1,30),NA),

Incidence_Deaths_NA=ifelse(is.na(Incidence_Deaths),rpois(1,1),NA)) %>%

ungroup -> Df

(K = nrow(Df))

# Convolutions = calculation of initial values for MCMC simulations

# that are used as initial values for the followed inference

incubation_shape = 8.038

incubation_rate = 1/0.2278

death_shape = 3.3012

death_rate = 1/0.5968

incubation_probability = pgamma(1:K,shape=incubation_shape,rate=incubation_rate)-pgamma(1:K-1,shape=incubation_shape,rate=incubation_rate)

timeFromOnsetToDeath_probability = pgamma(1:K,shape=death_shape,rate=death_rate)-pgamma(1:K-1,shape=death_shape,rate=death_rate)

# time from Exposure event to Death is the convolution of two latter probabilities

timeFromExposureToDeath_probability = c(0)

for (x in 2:K) {

timeFromExposureToDeath_probability = c(timeFromExposureToDeath_probability,

sum(incubation_probability[1:(x-1)]*timeFromOnsetToDeath_probability[(x-1):1]))

}

### Machinery for Nimble

# Convolution functions

nimConvolutionWithCFR = nimbleFunction(

run = function(a = double(1), b = double(1), q = double(1)) {

L <- dim(a)[1]

res1 <- numeric(L)

for(k in 1:L) {

res1[k] <- q[k]*a[k]

}

ans <- inprod(res1[L+1-1:L],b)

return(ans)

returnType(double(0))

}

)

nimConvolution = nimbleFunction(

run = function(a = double(1), b = double(1)) {

L <- dim(a)[1]

ans <- inprod(a[L+1-1:L],b)

return(ans)

returnType(double(0))

}

)

## Shift in l1 and l2 used to avoid edge effects

shift = 26

nCases = length(onsetTimes)

## Main script

nimData = list(# incubation and period to death

onsetTime = onsetTimes,

deathTime = deathTimes,

# incidence

infected = Df$Incidence_Reported,

dead = Df$Incidence_Deaths,

# for contraint

one = 1

)

# the following values are used for priors

incubation_mean_median = 13.16830

incubation_mean_lower = 10.99341

incubation_mean_upper = 15.52661

incubation_sd_median = 5.257898

incubation_sd_lower = 3.530271

incubation_sd_upper = 7.312072

death_mean_median = 14.21680

death_mean_lower = 10.83839

death_mean_upper = 17.70390

death_sd_median = 8.033101

death_sd_lower = 5.157413

death_sd_upper = 11.476860

nimConsts = list(nCases = length(onsetTimes),

incubation_uncertainty = 0.5,

death_uncertainty = 0.5,

exposure_mu = .5*(exposureTimesLower+exposureTimesUpper),

exposure_sd = (exposureTimesUpper-exposureTimesLower)/1.96/2,

r = rho,

K = K,

week = (Df$Week-shift-1)%%52+1,

incubation_mean_mean = (incubation_mean_upper+incubation_mean_lower)/2,

incubation_mean_sd = (incubation_mean_upper-incubation_mean_lower)/1.96/2,

incubation_sd_mean = (incubation_sd_upper+incubation_sd_lower)/2,

incubation_sd_sd = (incubation_sd_upper-incubation_sd_lower)/1.96/2,

death_mean_mean = (death_mean_upper+death_mean_lower)/2,

death_mean_sd = (death_mean_upper-death_mean_lower)/1.96/2,

death_sd_mean = (death_sd_upper+death_sd_lower)/2,

death_sd_sd = (death_sd_upper-death_sd_lower)/1.96/2

)

nimInits = function(){

lambdaIncidence0 = rexp(K,1/3); CFR0 = runif(K,0.02,0.12);

list(# incubation and period to death

incubation_mean = runif(1,incubation_mean_lower,incubation_mean_upper),

incubation_sd = runif(1,incubation_sd_lower,incubation_sd_upper),

death_mean = runif(1,death_mean_lower,death_mean_upper),

death_sd = runif(1,death_sd_lower,death_sd_upper),

onsetExpectedTime = onsetTimes,

deathExpectedTime = deathTimes,

exposureTime = .5*(exposureTimesLower+exposureTimesUpper),

incubationTime = onsetTimes-.5*(exposureTimesLower+exposureTimesUpper),

timeToDeath = deathTimes-onsetTimes,

# results

incubationPeriod = incubation_probability,

timeFromOnsetToDeath = timeFromOnsetToDeath_probability,

timeFromExposureToDeath = timeFromExposureToDeath_probability,

# incidence

l1 = (runif(1,44,52)-shift-1)%%52+1,

l2 = (runif(1,5,12)-shift-1)%%52+1,

infected = Df$Incidence_Reported_NA,

dead = Df$Incidence_Deaths_NA,

lambdaIncidence = (1-CFR0)*lambdaIncidence0,

lambdaDeath = CFR0*lambdaIncidence0,

lambdaReported = lambdaIncidence0,

pDeath = CFR0,

exposure = lambdaIncidence0,

CFR = CFR0,

mean_a = c(runif(1,5,15),runif(1,.5,3)),

sd_a = runif(2,0.1,1),

mean_q = runif(1,0.02,0.12),

sd_q = runif(1,0.1,1))}

nimCode = nimbleCode({

### incubation period and period from illness onset to death

incubation_mean ~ dnorm(mean=incubation_mean_mean, sd=incubation_mean_sd)

incubation_sd ~ dnorm(mean=incubation_sd_mean, sd=incubation_sd_sd)

death_mean ~ dnorm(mean=death_mean_mean, sd=death_mean_sd)

death_sd ~ dnorm(mean=death_sd_mean, sd=death_sd_sd)

for (k in 1:nCases) {

exposureTime[k] ~ dnorm(mean=exposure_mu[k],sd=exposure_sd[k])

incubationTime[k] ~ dgamma(mean=incubation_mean, sd=incubation_sd)

timeToDeath[k] ~ dgamma(mean=death_mean, sd=death_sd)

onsetExpectedTime[k] <- exposureTime[k] + incubationTime[k]

deathExpectedTime[k] <- onsetExpectedTime[k] + timeToDeath[k]

onsetTime[k] ~ dnorm(onsetExpectedTime[k], sd=incubation_uncertainty)

deathTime[k] ~ dnorm(deathExpectedTime[k], sd=death_uncertainty)

}

### epidemiological model

incubation_shape <- incubation_mean^2/incubation_sd^2

incubation_rate <- incubation_mean/incubation_sd^2*7.0 #from days to weeks

death_shape <- death_mean^2/death_sd^2

death_rate <- death_mean/death_sd^2*7.0 #from days to weeks

for (k in 1:K) {

incubationPeriod[k] <- pgamma(k,shape=incubation_shape,rate=incubation_rate)-pgamma(k-1,shape=incubation_shape,rate=incubation_rate)

timeFromOnsetToDeath[k] <- pgamma(k,shape=death_shape,rate=death_rate)-pgamma(k-1,shape=death_shape,rate=death_rate)

}

timeFromExposureToDeath[1] <- 0

for (k in 1:(K-1)) {

timeFromExposureToDeath[k+1] <- nimConvolution(incubationPeriod[1:k],timeFromOnsetToDeath[1:k])

}

### exposure

for (k in 1:K) {

mean_a_realized[k] <- mean_a[1]+(mean_a[2]-mean_a[1])*equals(step(week[k]-l1),step(l2-week[k]))

sd_a_realized[k] <- sd_a[1]+(sd_a[2]-sd_a[1])*equals(step(week[k]-l1),step(l2-week[k]))

exposure[k] ~ dgamma(mean=mean_a_realized[k],sd=sd_a_realized[k])

CFR[k] ~ dgamma(mean=mean_q,sd=sd_q)

}

for (k in 52:(K-1)) {

lambdaIncidence[k+1] <- nimConvolutionWithCFR(exposure[1:k],incubationPeriod[1:k],1-CFR[1:k])/(1-r)

lambdaDeath[k+1] <- nimConvolutionWithCFR(exposure[1:k],timeFromExposureToDeath[1:k],CFR[1:k])/(1-r)

lambdaReported[k+1] <- lambdaDeath[k+1]+lambdaIncidence[k+1]

pDeath[k+1] <- lambdaDeath[k+1]/lambdaReported[k+1]

}

for (k in 53:K) {

infected[k] ~ dpois(lambdaReported[k])

dead[k] ~ dbin(pDeath[k],infected[k])

}

## Priors

for(i in 1:2) {

mean_a[i] ~ dhalfflat()

sd_a[i] ~ dhalfflat()

}

mean_q ~ dhalfflat()

sd_q ~ dhalfflat()

l1 ~ dunif(0,53)

l2 ~ dunif(0,53)

one ~ dconstraint(l2>l1-3)

})

nimModel = nimbleModel(nimCode,

constants = nimConsts,

data = nimData,

inits = nimInits())

## Checking that all variables are properly initialized

nimModel$initializeInfo()

nimConf = configureMCMC(nimModel, thin = 100, setSeed=TRUE)

nimConf$addMonitors(c("incubationTime","timeToDeath","lambdaIncidence","lambdaDeath","pDeath","exposure","CFR","infected","incubationPeriod","timeFromOnsetToDeath","timeFromExposureToDeath"))

## Model compilation

nimMCMC = buildMCMC()

compiledModel = compileNimble(nimModel, nimMCMC, resetFunctions = TRUE, showCompilerOutput = TRUE)

Niter = 1e5

Nburn = 2e4

compiledModel$nimMCMC$run(niter=Niter+Nburn, nburnin = Nburn)

compiledModel$nimMCMC$mvSamples %>% as.matrix %>% as.data.frame -> nimSamples

saveRDS(nimSamples, file = paste0("nimSamples-",args[1],".rds"))

compiledModel$nimMCMC$getTimes() %>% { sum(.)/60 }

**Appendix Code Snippet 2: An example of a bash script applied to Appendix Code Snippet 1 “MCMC.r”**

#!/bin/bash

for replicate in {1..25}

do

R CMD BATCH --no-save --no-restore '--args '"${replicate}" MCMC.r MCMC-final-${replicate}.Rout &

done
